# Daily relationship between air pollution and physical activity in an industrial region

**DOI:** 10.1111/aphw.70186

**Published:** 2026-07-07

**Authors:** Lenka Knapová, Radim Lískovec, Young Won Cho, Dario Baretta, Jan Keller, Steriani Elavsky

**Affiliations:** ^1^ Department of Human Movement Studies University of Ostrava Ostrava Czech Republic; ^2^ Department of Geography Masaryk University Brno Czech Republic; ^3^ Department of Human Development and Family Studies The Pennsylvania State University University Park Pennsylvania USA; ^4^ Institute of Psychology University of Bern Bern Switzerland; ^5^ Department of Psychology Heidelberg University Heidelberg Germany; ^6^ Department of Education and Psychology Freie Universität Berlin Berlin Germany

**Keywords:** air pollution, climate change, environmental exposure, intensive longitudinal data (ILD), physical activity, weather

## Abstract

Physical activity (PA) is essential for physical and mental health, yet remains insufficient across populations. Environmental factors, such as air pollution (AP), are increasingly recognized as dynamic influences on daily PA behavior. This study investigated within‐person relationships between AP (particulate matter PM₁₀) and device‐based PA (step counts) over 14 days in a sample of 693 adults living in an industrial region in the Czech Republic (9702 daily observations; 47.2% women; Mage = 38.78, SD = 12.28). The sample was recruited using non‐probabilistic quota sampling. Using multilevel Bayesian modeling, we found that on days when PM₁₀ was higher than a participant's average across the 14 days, step counts were lower (*b* = −0.027; 95% CI = [−0.047, −0.006]), while controlling for the effects of temperature, sunshine, and precipitation. Specifically, a 1‐SD increase in PM₁₀ was linked to 361 fewer steps. Self‐reported AP monitoring tendency did not moderate the within‐person relationship between AP and step counts (*b* = −0.003; 95% CI = [−0.044, 0.038]). Findings highlight the importance of capturing short‐term environmental exposures in PA research and underscore the public health relevance of behavioral adaptation under conditions shaped by climate change, including AP. Results may inform individual‐level digital interventions focused on adaptive behavior change as well as policy‐level urban planning strategies that promote PA under varying environmental conditions.

## INTRODUCTION

Regular physical activity (PA) is fundamental to physical and mental health, with sustained PA linked to lower all‐cause mortality, reduced risk of cardiovascular and chronic diseases (Ekelund et al., 2019; World Health Organization [WHO], 2020), and improved mental health and well‐being (Marquez et al., 2020; McDowell et al., 2019; Pearce et al., 2022; WHO, 2020). Despite these well‐established benefits, a large proportion of the global population fails to meet the recommended PA levels (Guthold et al., 2018; WHO, 2022).

Research seeking to explain PA behavior has traditionally emphasized individual‐level and psychosocial determinants, while environmental determinants have received comparatively less attention. When included, they have typically been conceptualized as social environmental factors such as social norms, social support, or social cues (Bandura, 1986; Luszczynska & Schwarzer, 2005; Rosenstock, 1990). Only a few behavioral models explicitly extend beyond the social domain. Specifically, the COM‐B model acknowledges physical opportunity as a contributor to behavior (Michie et al., 2011), and ecological models highlight multiple levels of influence, including also the natural and built environment more generally (Sallis et al., 2008). Nevertheless, applications of ecological models in PA research have largely focused on relatively stable built‐environment characteristics such as walkability, land use, greenery, and neighborhood design (e.g., McCormack & Shiell, 2011; Saelens & Handy, 2008), with fewer studies considering dynamic environmental exposures. In this study, we adopt a broad ecological perspective, in which environmental exposures are considered alongside psychological‐level determinants of daily PA, with potential interactions across levels (Sallis et al., 2008).

Recently, dynamically changing environmental risk exposures, including air pollution (AP) and weather conditions, have increasingly been examined in relation to PA behaviors (e.g., An et al., 2018; Ferguson et al., 2023). This emerging focus is driven both by improved environmental monitoring technologies and by heightened concern about climate‐related risks, including rising temperatures, extreme weather events, and persistent AP in many urban regions (Bernard et al., 2021; European Environment Agency [EEA], 2022; Fuller et al., 2022). Despite recent improvements in air quality, particularly in high‐income regions such as North America and parts of Europe (EEA, 2024; U.S. Environmental Protection Agency, 2025), AP remains a major public health concern with 99% of the world's population living in areas exceeding WHO's limits (WHO, 2023). The EEA (2024) continues to identify AP as a major environmental health risk, with 239,000 deaths in the European Union countries in 2022 attributable to exposure to particulate matter (PM) concentrations above the WHO guideline level. Particulate matter consists of inhalable solid particles and liquid droplets suspended in the air. Importantly, exceedances of air quality standards or AP levels are observed across various regions of Europe, including hotspots in both highly developed and structurally disadvantaged areas. However, populations residing in lower‐income regions, often located in Central and Eastern Europe, are more vulnerable to adverse health effects of AP (Clayton et al., 2025; Richardson et al., 2013).

Beyond the well‐supported direct negative influence of AP on individuals' health (e.g., Dominski et al., 2021), AP may also be associated with changes in health behaviors, including PA. A growing body of evidence (see reviews by An et al., 2018, 2019; Kim et al., 2021; Tainio et al., 2021; and Bernard et al., 2021) suggests that higher AP levels are associated with reduced PA, including shorter durations of outdoor leisure‐time and transportation‐related PA, higher odds of leisure‐time inactivity, and potentially stronger effects among vulnerable populations. Nevertheless, these reviews note that the majority of the reviewed studies were cross‐sectional and geographically concentrated, particularly in China and the United States (An et al., 2018, 2019; Kim et al., 2021; Tainio et al., 2021).

Given that AP changes dynamically over time, methods enabling repeated, real‐world behavioral assessment are needed to capture how day‐to‐day exposure relates to day‐to‐day PA. Ambulatory assessment approaches, including ecological momentary assessment (EMA) and continuous device‐based monitoring, allow frequent measurement of behavior and context in participants' environments, reduce recall bias, and enable investigation of both within‐ and between‐person variability (Reichert et al., 2020; Trull & Ebner‐Priemer, 2013). Reflecting these advantages, an emerging body of evidence has examined within‐person associations by using wearable devices to capture PA and linking these data with daily or hourly AP measures.

For instance, Alahmari et al. (2015) monitored 73 chronic obstructive pulmonary disease patients in central London, UK, for 29–658 days using accelerometers, finding that higher PM₁₀ and ozone levels obtained from a single monitoring site were associated with decreased PA, particularly on weekdays. Yu et al. (2021) collected continuous accelerometer data over 7 days from 340 university students in Beijing, showing negative hourly associations of PM_2.5_ and overall air quality index with moderate‐to‐vigorous PA and step counts. Similar to Alahmari et al. (2015), AP data in this study were obtained from a single location, here an AP station about 5 km from the university campus (Yu et al., 2021). Cheng et al. (2024) measured PA over seven consecutive days in 184 older adults from three Chinese cities using accelerometers and linked AP data from monitoring stations close to participants' addresses—one for each city. They observed negative associations of air quality index and PM_2.5_ with daily walking, light, moderate, and moderate‐to‐vigorous PA, while PM₁₀ was negatively related only to light activity. Recently, two additional studies were conducted with patients after bariatric surgery and chronic obstructive pulmonary disease patients. In a bariatric surgery cohort of 77 US patients, Baillot et al. (2024) found that higher air quality index was associated with lower accelerometer‐based daily light and moderate‐to‐vigorous PA and greater sedentary time before and after surgery. AP and weather data were obtained from a single station based on participants' city of residence (Baillot et al., 2024). Finally, a multi‐center panel study of 408 chronic obstructive pulmonary disease patients in Catalonia, Spain, measured daily PA over two 7‐day periods with accelerometers and found that higher AP (NO_2_, PM₁₀, absorbance of PM_2.5_ estimated for participants' residential addresses) was linked with reduced steps, lower MVPA, and increased sedentary time over the following days (Josa‐Culleré et al., 2024).

The above‐mentioned intensive longitudinal studies have provided valuable insights into how daily AP influences PA, but methodological differences remain important to consider. Many studies relied on single AP monitoring stations, without accounting for individuals' daily mobility and potential variation in exposure, and often utilized short monitoring periods or samples drawn from specific populations, predominantly in China and in a few high‐income countries. Importantly, previous research has indicated that findings from one region or country may not be generalizable to others because of substantial global variability in AP sources, population vulnerability, and PA patterns (Tainio et al., 2021). Even within the same city, pollution levels can differ dramatically between city zones (Nieckarz & Zoladz, 2020). Thus, more research is needed across diverse countries and regions. At the same time, although these studies suggest that higher AP is associated with lower PA, the mechanisms underlying this relationship remain less clearly understood.

Several plausible pathways may help explain why short‐term increases in AP coincide with reduced PA. Acute exposure to particulate and gaseous pollutants can lead to respiratory symptoms (e.g., coughing, shortness of breath), systemic symptoms (e.g., headaches, dizziness) as well as lung function decline and increased inflammatory markers, making PA and active transportation uncomfortable or more effortful (e.g., Cutrufello et al., 2011; Dauchet et al., 2018; Dominski et al., 2021; Kocot & Zejda, 2021; Manisalidis et al., 2020). Additionally, short‐term exposure to AP has been associated with reduced cognitive performance and impaired decision‐making (e.g., Aguilar‐Gomez et al., 2022; Shehab & Pope, 2019), which may indirectly contribute to lower PA by diminishing the cognitive resources required for planning or sustaining activity. Individuals may also perceive (e.g., see or smell) the AP (Deguen et al., 2012; Noël et al., 2022) and respond behaviorally by postponing or shortening outdoor activity, but studies focusing explicitly on the link between sensory perception of AP and PA are lacking.

Nevertheless, AP is not always directly perceptible, and individuals often need to rely on external information sources, such as air quality apps or media alerts, to judge exposure risk and adapt behaviors. This makes understanding how AP monitoring contributes to behavioral responses an important area of research. To complement the ecological perspective, the Protection Motivation Theory (PMT; Rogers, 1983) provides a useful framework for understanding one further possible pathway through which individuals respond to environmental threats such as AP. PMT posits that threat appraisal, including the evaluation of threat severity and one's vulnerability to it, can motivate protective behavior. In this context, monitoring environmental information such as generally following AP updates may serve as a proxy for heightened threat appraisal and could indicate greater readiness to adjust behavior on high‐pollution days. Consequently, individuals who regularly follow AP information may be more likely to reduce PA on high‐pollution days.

Empirical evidence linking AP monitoring, awareness, or alert exposure to changes in PA remains limited. A systematic review by D'Antoni et al. (2017) showed that adherence to recommended protective behaviors following air‐quality alerts is generally modest (a median of 31% for reducing outdoor activities) and varies with individual factors such as perceived severity, symptom attribution, and knowledge of where to check air‐quality information. Further, reduced cycling traffic on polluted days was also observed when air‐quality alerts were issued (Saberian et al., 2017), and real‐time pollution information was linked to lower attendance at outdoor sporting events (Yoo, 2021). A survey‐based study also found that individuals who reported following air‐quality information were more likely to modify their outdoor activity on poor‐air‐quality days (Wen et al., 2009), and device‐based tracking suggests reduced running when pollution warnings appear on mobile platforms (Yang et al., 2024). However, these studies are largely correlational and therefore cannot determine directionality or causality. While this body of work supports the idea that AP information may play a role in behavioral adjustment, it does not clarify whether monitoring behavior reflects underlying threat appraisal, contributes to it, or both. Examining AP monitoring as a potential moderator of the daily AP–PA association therefore remains theoretically motivated but requires empirical testing.

Regardless of the specific mechanisms involved, investigating behavioral responses to AP also requires accounting for other environmental influences, particularly weather. Most of the previously discussed intensive studies evaluating the link between AP and PA have accounted for weather exposure variables, recognizing that AP and weather conditions co‐occur and can jointly influence daily PA. In addition, a broader body of research has independently examined how weather relates to daily PA. Across both lines of evidence, temperature generally shows a positive association with PA (Alahmari et al., 2015; Chan et al., 2006; Josa‐Culleré et al., 2024; Timm et al., 2023; Turrisi et al., 2021), although emerging evidence indicates nonlinear associations at higher temperature ranges, with (extreme) heat linked to reduced activity (Baillot et al., 2024; Ferguson et al., 2023; Ho et al., 2022). Other weather indicators, such as longer sunshine duration (Alahmari et al., 2015; Ferguson et al., 2023; Klenk et al., 2012) and lower precipitation (Alahmari et al., 2015; Chan et al., 2006; Ferguson et al., 2023; Josa‐Culleré et al., 2024; Turrisi et al., 2021), have also been associated with higher PA. Accordingly, weather variables represent essential covariates when modeling the daily AP‐PA associations.

### Present study

Building on prior research, the present study addresses several important limitations in the existing literature by examining daily associations between AP and PA within individuals living in a highly industrialized region in the Czech Republic, Central Europe, a geographical context underrepresented in previous work. A further methodological contribution lies in assigning environmental exposure estimates based on participants' approximate daily locations using smartphone‐derived proxies. By linking these proxy locations to the nearest background monitoring stations, this approach provides a more spatially sensitive estimate of background AP exposure for each participant than relying on a single fixed monitoring station for the entire sample, as has been common in previous studies.

The present study utilizes data from a 14‐day intensive monitoring burst of a larger prospective study. The primary aim of the present study is to investigate whether daily PA (step counts) co‐varies with daily AP (PM₁₀) levels at the within‐person level. We hypothesize that on days when PM₁₀ is above the 14‐day average for a given participant, their daily step counts are lower.

Additionally, to examine whether responses to AP vary across individuals, we evaluate general AP monitoring tendency as a cross‐level moderator. We hypothesize that individuals who generally follow AP information will show stronger reductions in daily PA on high‐pollution days compared to those who do not follow AP information. Daily weather conditions (air temperature, precipitation, and sunshine duration) are included as important covariates given their established associations with day‐to‐day variability in PA.

## METHODS

### Study procedure

The present study uses data from the Healthy Aging in Industrial Environment (4HAIE) study, a 12‐month prospective longitudinal study designed to explore the links among biomechanical, physiological, psychosocial, sociodemographic, and environmental variables, and their interaction on physical (in)activity, health (including running‐related injuries), and quality of life among adults aged 18 to 65 years. A prospective cohort of 1314 participants was established in two Czech regions with contrasting ambient AP: the Moravian–Silesian Region (higher AP, industrial area; *n* = 750) and the South Bohemian Region (lower AP; *n* = 564). Despite their relative geographical proximity and climatological similarities, the two regions differ markedly in annual mean concentrations and long‐term exposure to air pollutants such as PM₁₀, PM_2.5_, NO_2_, benzene, and benzo(a)pyrene (CHMI, 2021; Machaczka et al., 2023).

The various data streams that were collected in the 4HAIE study are described in the respective study protocols (Cipryan et al., 2020; Elavsky et al., 2021; Jandacka et al., 2020) and can be found on the study website: https://haie-lerco.cz/en/home/. Several original articles have been published based on the 4HAIE study data (see here: https://haie-lerco.cz/en/https-haie-lerco-cz-en-publications-papers/papers/).

The study procedures were approved by the Ethics Committee of the University of Ostrava (No. OU‐22953/90–2020), and participants provided written informed consent prior to data collection, adhering to the standards set by the Declaration of Helsinki.

The participants were recruited by a professional social science research and marketing company selected through a public tender. Recruitment strategies included online outreach (e.g., social media posts, fora, job platforms), community‐based efforts at local venues and events (e.g., sports organizations, shopping malls), media advertisements (local newspapers, public transit, radio), the company's interviewer network, and chain referral. Eligibility was screened via an online survey and a follow‐up telephone interview. Inclusion criteria were age 18–65 years, non‐smoker, access to a smartphone (Android or iOS operating systems) with Internet, and no physician‐diagnosed restrictions to PA. Exclusion criteria included pregnancy, acute illness at baseline testing, contraindications to magnetic resonance imaging, or conditions limiting PA (Elavsky et al., 2021).

The sample was recruited using non‐probabilistic quota sampling based on age, gender, PA status, and region. Age and gender quotas reflected the general population distributions. For PA status, the goal was to recruit approximately 60% active runners and 40% insufficiently active adults. Active runners were defined as those who met PA guidelines (≥150 min/week of MVPA) and reported regular running of at least 10 km/week for ≥6 weeks; inactive participants reported <150 min/week of MVPA but were capable of running. Quotas were continuously monitored in the screening survey; once a quota was filled (e.g., men aged 35–44 from the Moravian–Silesian Region), individuals with those characteristics were no longer enrolled.

Data were collected between April 2019 and September 2022. The participants attended baseline laboratory testing at the university, after which they entered a 12‐month ambulatory assessment monitoring phase. During this period, they wore a Fitbit Charge 3 (or Charge 4 for participants recruited in later stages of the study). The participants also went through four 14‐day bursts (baseline, month 4, month 8, month 12) of EMA, with four short smartphone surveys per day delivered at semi‐random times within fixed time windows.

#### Present study data

The present analyses focus on a subset of 4HAIE data: the first 14‐day monitoring burst (weeks 1–2 of the study), which occurred between April 2019 and September 2021 depending on each participant's study entry date. Analyses were restricted to participants residing in the Moravian–Silesian Region only. The Moravian–Silesian Region is an industrial region in eastern Czechia with comparatively higher background AP (CHMI, 2021) and denser air‐quality monitoring coverage than the South Bohemian Region, with 16 background stations in Moravia–Silesia versus five in South Bohemia. In addition to AP exposure, the Moravian–Silesian Region faces long‐term socioeconomic disadvantages, which may increase population vulnerability to the health effects of AP (Richardson et al., 2013). Given the aims of this study to examine within‐person associations between daily AP and device‐based PA, we restricted analyses to participants residing in the Moravian–Silesian Region to focus on a highly polluted setting with clear public‐health relevance and to maximize the spatial accuracy of exposure estimates by leveraging the denser monitoring network.

We restricted analyses to the first 14‐day burst for methodological reasons, specifically to ensure high‐quality environmental exposure linkage. Assigning daily AP and weather exposure depended on smartphone‐based location data, which were collected by the study survey app (for Android and iOS). During the in‐person onboarding, study staff confirmed correct location permissions, minimized battery‐optimization restrictions, and verified app behavior for accurate location retrieval. In later stages of the 12‐month monitoring, the participants could modify settings (intentionally or automatically), leading to increased missingness and poorer spatial accuracy of location‐based exposure estimates. As is typical for longitudinal digital studies, adherence also declined over time. The first burst showed the highest survey app usage, and therefore the highest density of valid location records, making it the most reliable burst for the environmental exposure‐mapping approaches used here (see location‐based data).

**FIGURE 1:**
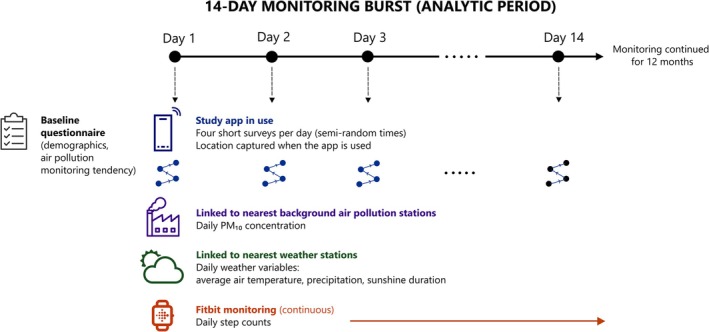
Overview of the 14‐day monitoring burst and data streams used in the present study.

Thus, the present study utilizes (a) Fitbit‐derived step counts from the first 14‐day burst, (b) related daily environmental exposure estimates linked to participants' proxy locations, and (c) baseline questionnaire data (see Figure 1 for an overview of the present study timeline and data streams).

### Participants

Of the total 4HAIE cohort, *N* = 750 participants resided in the Moravian–Silesian Region and were initially eligible for the present study. These participants met the inclusion criteria described earlier and completed baseline assessments and the first 14‐day monitoring burst.

After applying data‐quality criteria to PA and location data (see Missing data and data quality), the final analytic sample comprised *n* = 693 participants.

### Measures

Several streams of data were combined in this study (see Figure 1). PA data were derived from the Fitbit Charge fitness monitors. The participants reported demographic variables and general AP monitoring tendency in the online baseline questionnaire. Additionally, we made use of participants' proxy location information and combined these data with validated measurements from the Czech Hydrometeorological Institute (CHMI) on background AP and weather.

#### Demographic data

In the baseline survey, we asked for basic demographic information, including age, gender (1: male; 0: female), and university education. Education was originally reported on a 5‐point scale from primary, through high school, to university education. It was recoded into a binary variable (1: university education, 0: no university education).

With respect to PA status, the participants were labelled as either runners (1) or inactive participants (0) at baseline based on self‐reported PA data. Runners were participants who self‐reported meeting PA guidelines of 150 MVPA per week or more and engaging in regular running for at least 6 weeks at the level of 10 km per week or more in the baseline online screening survey verified in subsequent telephone screening. Inactive participants indicated engaging in PA at levels lower than PA guidelines but were capable of running.

#### PA data

The participants were monitored by a Fitbit Charge 3 device (or Charge 4 for participants in the later parts of the study because of unavailability of the Charge 3 version to replace faulty devices) and were instructed to wear the device on the non‐dominant wrist continuously throughout the day, including sedentary periods and sleep, except during activities where wearing a watch could pose a safety risk (e.g., climbing, sauna). The Fitbit Charge 3 and Charge 4 used in this study are highly comparable in hardware (both use a tri‐axial accelerometer) and step‐counting algorithms (validation evidence summarized in Feehan et al. [2018] and Henriksen et al. [2018]). Step counts were selected because they provide a scalable, unobtrusive, and time‐sensitive indicator of daily movement patterns, making them well suited for continuous monitoring in real‐world settings (Baretta et al., 2025). Although they do not capture all dimensions of PA, such as activity intensity or non‐stepping types of activities, they are readily interpretable and have been linked to important health outcomes in recent meta‐analytic evidence (Ding et al., 2025; Paluch et al., 2022). While some research suggests that Fitbit devices may overestimate steps in free‐living settings (Feehan et al., 2018), they provided the most feasible and scalable option for continuous monitoring in the present study. Step‐count data were automatically synchronized to the Fitbit cloud and securely transferred to the HealthReact study server (HealthReact, n.d.).

##### Fitbit wear time

Total daily minutes of non‐sleep Fitbit wear were calculated and subsequently re‐coded as hours/day. A valid Fitbit wear minute was defined as a non‐sleep minute where either at least one heart rate value or non‐zero step counts were recorded.

##### Daily step counts

Daily PA was assessed as total daily step counts by the Fitbit Charge 3 or 4 device. Fitbit devices use a proprietary algorithm for step detection and do not provide access to raw sampling frequency or filtering parameters. Fitbit devices provide processed minute‐level step count data. The total number of steps per day was calculated as the sum of the non‐sleep minute‐level values.

#### Location‐based data

In the present study, the location information was obtained when participants interacted with the study survey app (e.g., completed an EMA survey). At each app interaction, the participant was assigned to the nearest air quality monitoring station within the CHMI network, regardless of station type (e.g., background, traffic, or industrial hotspot). To protect privacy, the participants' exact coordinates were not stored. Instead, the app recorded the longitude and latitude of the nearest station and the distance between the participant and that station at the time of the interaction.

Operational air quality data from these nearest stations were available in near real‐time and stored by the researchers. However, these data were available only as point‐in‐time operational values that could not be reliably aggregated into daily averages, were often incomplete, and had not yet undergone validation. In addition, many nearest stations measured only a limited subset of pollutants or represented highly localized traffic or industrial hotspots with limited spatial representativeness for daily exposure assessment.

**FIGURE 2:**
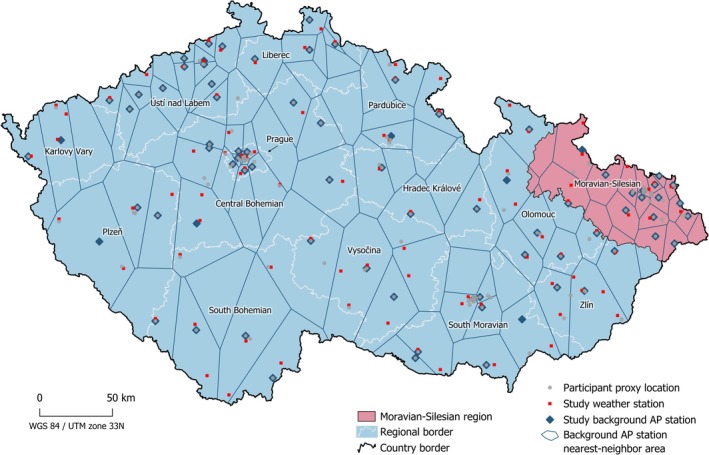
Participant proxy locations (nearest air pollution stations), subset of background air pollution (AP) stations, and weather stations specifically associated with the study participants, including nearest‐station service areas for all background stations in the Czech Republic, with the Moravian–Silesian Region highlighted (region of residence of participants). *Not*e: Station positions varied during the study period; the configuration presented corresponds to the year 2021.

Therefore, these unvalidated operational data were not used for the present analyses, and the nearest‐station assignments were used primarily as location proxies. Each proxy location was subsequently linked to validated daily AP concentrations from the nearest representative background monitoring station (urban, suburban, rural, or regional) and to validated weather data from the nearest weather station (see Figure 2). To ensure spatial consistency, we applied quality filters to exclude records with low location accuracy, excessive distance to the air quality monitoring station or the nearest representative background station, and unusually large daily mobility. Additional methodological details are provided in Data S7.

### Particulate matter 10 (PM₁₀)

AP was assessed using concentrations of inhalable particulate matter with an aerodynamic diameter of 10 μm or less (PM₁₀). Validated daily PM₁₀ concentrations (μg/m^3^; Open Data CHMI, 2025) were linked from the nearest background AP stations (see study background AP stations in Figure 2) to participants' proxy locations. The participant‐specific PM₁₀ daily value was calculated as the mean of the daily PM₁₀ concentrations for all the proxy location records for a given participant on a given day.

#### Note on AP parameter selection

While PM_2.5_ is often prioritized in recent research, the present study used PM₁₀ because of its higher data availability and regulatory relevance. Within the Czech national monitoring network, PM₁₀ is monitored at more stations and with fewer data gaps, ensuring more reliable and spatially consistent exposure estimates. PM₁₀ is also historically central to air quality assessment because it reflects key emission sources (e.g., residential heating, resuspension). Czech legislation explicitly sets both a daily (24‐h) limit of 50 μg/m^3^ for PM₁₀ (permitted to be exceeded 35 times per year) and an annual limit of 40 μg/m^3^, whereas PM_2.5_ is regulated only by an annual limit of 20 μg/m^3^ (Act No. 201/2012 Coll. Act on Air Protection; Czech Republic, 2012). Moreover, Czech national air pollution alerts, smog regulations, and the air quality index are all based on PM₁₀ threshold values. For these reasons, PM₁₀ provides both robust empirical coverage and direct policy relevance for the present analysis.

For each participant's proxy location, validated weather data (Open Data CHMI, 2025) from the nearest weather station were used (see study weather stations in Figure 2). When data for a specific day or variable were missing in the validated CHMI dataset, the value from the next nearest weather station was substituted.

These variables follow the standard meteorological metrics and calculation conventions used in official CHMI climatological reporting.

### Average daily air temperature

The average air temperature (°C) is calculated by the CHMI as the mean of the measurements taken at 7 a.m., 2 p.m., and 9 p.m. h local mean solar time, with the 9 p.m. measurement counted twice. The participant‐specific average daily air temperature was calculated as the mean of the average air temperatures for all the proxy location records for a given participant on a given day.

### Daily precipitation

Daily precipitation (millimeters) refers to the total amount of precipitation that falls within a 24‐h period. It is measured at 7 a.m. local mean solar time and assigned to the previous day by the CHMI. The participant‐specific daily precipitation was calculated as the mean of the daily precipitation for all the proxy location records for a given participant on a given day.

### Daily sunshine duration

The daily duration of sunshine (cumulative hours) is provided by the CHMI as the time between sunrise and sunset during which the sun is not obscured by clouds or other obstructions. Physically, it corresponds to periods when the direct solar radiation flux exceeds 120 W/m^2^. Over the course of the year, sunshine duration follows the pattern of theoretical sunshine length and cloud cover variability; on a daily scale, it is primarily influenced by cloudiness. The participant‐specific daily sunshine duration was calculated as the mean of the daily sunshine hours for all the proxy location records for a given participant on a given day.

#### AP monitoring tendency (general)

In the baseline survey (i.e., reported only once at the beginning of the study), the participants indicated whether they generally keep track of current AP levels using a single yes (1)/no (0) question *Do you follow current information on the state of air pollution?* This item reflects a self‐reported, general tendency to monitor AP information.

### Missing data and data quality

To ensure data quality, we defined a valid Fitbit day as ≥10 waking hours of device wear time (Troiano et al., 2008) and a non‐zero step count. Days with zero steps were treated as missing. The participants were required to have a minimum of three valid (non‐missing) step count days (Yao et al., 2021) for inclusion in the analysis.

Of the 750 participants residing in the Moravian–Silesian Region, 39 did not provide any smartphone‐based location data, preventing the calculation of AP and weather exposures; these participants were excluded. An additional 14 participants were excluded because they had fewer than three valid stepcount days, and four participants were excluded because of missing data on binary exogenous variables (university education and AP monitoring tendency). The final analytic sample thus consisted of *n* = 693 participants.

In the final dataset, step count data had an average missing rate of 8.84% per individual over the 14‐day period (median = 0%), corresponding to an average of 12.77 available days (median = 14). AP data had an average missing rate of 15.2% (median = 7.1%), corresponding to an average of 11.88 available days per participant (median = 13). Missing data were handled using Mplus's default approach for Bayesian estimator (Asparouhov & Muthén, 2010), which imputes missing values for endogenous variables (e.g., step counts) using model‐implied plausible values.

Regarding statistical power, Arend and Schäfer's (2019) simulation for multilevel studies with high intraclass correlation indicates that a sample of 200 individuals with 14 observations provides approximately 80% power to detect small within‐person effects (*b* = 0.09) and small‐to‐medium between‐person (*b* = 0.21) and cross‐level interaction effects (*b* = 0.23). With 693 participants, our study is expected to provide adequate power for detecting the effects of comparable magnitude. To supplement this, we conducted a post hoc Monte Carlo power analysis using our data structure and the observed variance components and unstandardized within‐person PM₁₀ slope estimated from our model (see Data S1). Simulating a two‐level random‐intercept–random‐slope model aligned with our analytic specification, the estimated power to detect the within‐person effect was 0.85.

### Data analyses

The data consisted of daily observations (Level 1: day‐level; within‐person) nested within individuals (Level 2: person‐level; between‐person). Given the repeated measurement structure (i.e., 14 days per individual), we calculated the intraclass correlation coefficient (ICC) for the step count outcome. The ICC was 0.38, indicating moderate within‐person clustering and supporting the use of multilevel modeling. Accordingly, we employed a two‐level model to examine both within‐ and between‐person associations between daily AP exposure (PM₁₀) and step count. The model was accounted for time‐varying contextual variables (e.g., temperature) and person‐level characteristics (e.g., age, gender, PA status, AP monitoring tendency).

#### Within‐person level model

At the within‐person level, daily step counts were predicted by the corresponding day's PM₁₀ concentration. This association was allowed to vary across individuals (i.e., random slope)—for example, some people might reduce their steps a lot on polluted days, while others might not change their behavior at all. To examine whether AP monitoring behavior moderates this relationship, we included a cross‐level interaction: the between‐person level indicator of whether participants generally follow AP information (i.e., AP monitoring tendency) was used to predict the person‐specific PM₁₀ effect.

At the within‐person (day) level, day‐level covariates were included to account for factors known to influence daily PA. These included daily weather conditions: air temperature, precipitation, and sunshine duration. Because there were 18 participants whose data collection overlapped with COVID‐19 lockdown in 2020, a binary variable indicating lockdown status (1 = lockdown day; 0 = non‐lockdown day) was added to account for potential mobility restrictions associated with the COVID‐19 pandemic. Additionally, daily wear time of the Fitbit activity tracker was included as a covariate to control for measurement bias because of incomplete data capture. All day‐level predictors were latent cluster mean‐centered, accounting for measurement error in cluster means (Asparouhov & Muthén, 2019). Random effects were estimated for the intercept and for the within‐person PM₁₀ effect. The full model equations are provided in the Data S1).

#### Between‐person level model

At the between‐person level, individuals' average step counts across 14 days were predicted by their average PM₁₀ concentrations. To examine the role of AP monitoring tendency, it was included as another predictor. An interaction term (PM₁₀ × AP monitoring tendency) was also added to test whether the association between PM₁₀ and step counts varied by the monitoring tendency. Person‐level covariates included age, gender, education, and self‐reported PA status. Between‐person covariates also included person‐level means of the weather variables, with the exception of temperature. The temperature mean was excluded because of multicollinearity concerns (VIF = 5.06); without it, all predictors had VIF values below 2. Between‐person covariates were grand‐mean centered.

#### Model estimation

Analysis was conducted using Mplus 8 (Muthén & Muthén, 2017). We employed Bayesian estimation for the multilevel model, which offers practical advantages when estimating complex multilevel models requiring integration over high‐dimensional parameter spaces (Cho et al., 2024; Oravecz et al., 2011). Unlike frequentist methods that produce single point estimates, Bayesian estimation yields a posterior distribution for each parameter, which is the updated probability distribution after combining prior beliefs with the observed data. Because Bayesian inference does not rely on asymptotic (large sample) theory, it is generally more robust to violations of normality assumptions (Muthén & Asparouhov, 2012). Following the recommendations for Bayesian analysis reporting (Kruschke, 2021), we report the median of the posterior distribution as the point estimate, along with its standard deviation and 95% credible intervals (CIs). Similar to the frequentist statistics, an effect is considered statistically significant if its 95% CI does not include zero. The Mplus analytical code and details on Mplus Bayesian estimation settings, including priors and convergence criteria, are provided in the Data S2 and S3.

## RESULTS

### Descriptive statistics

The final sample included *N* = 693 participants with 9,702 daily observations, consisting of 76% Android users and 24% iOS users. The average age of the sample was *M* = 38.78 (SD = 12.28, 18–65), and 47.2% (327) were women. At baseline, 57.7% (400) of participants categorized themselves as runners and 42.3% (293) as inactive individuals. Regarding family status, 58.7% (407) of the participants were married or cohabitating, 29.3% (203) were single, 10.8% (75) were divorced, and 1.2% (8) were widowed. The majority (79.9%, 551) rated their economic situation as average, 17.5% (121) as above average, and 2.6% (18) as below average. Slightly less than half of the participants (45.5%, 315) had tertiary/university education.

Observations were distributed across all seasons (Spring: 13.4%; Summer: 38.0%; Fall: 29.4%; Winter: 19.1%). On average, the participants recorded *M* = 13,934 steps per day (*SD* = 8,140) with an average Fitbit wear time of 15.41 h per day (*SD* = 2.57). While raw daily step counts showed the expected slightly right‐skewed distribution common in PA, the average within‐person skewness was 0.511 (IQR: −0.009 to 0.94), indicating that most individuals' step distributions were approximately Gaussian. The average daily PM₁₀ value was *M* = 21.72 (*SD* = 13.38) and ranged from 1.57 to 149.99. Average daily air temperature was *M* = 12.08 (*SD* = 7.66)°C, but it ranged widely from well below 0°C (−12.06°C) to a maximum temperature of 28.48°C. Hours of sunshine per day ranged from 0 to 15.3 h. Precipitation levels ranged from 0 to 82.17 mm per day. The proportion of days with zero precipitation was 53.6%.

Details on the descriptive statistics can be found in Table 1. The correlations among the key variables are presented in Table 1. The potential nonlinear effect of PM₁₀ on steps was explored using a generalized additive model, but the relationship was found to be approximately linear after controlling for weather variables. Full details are provided in the Data S5.

**TABLE 1:** Descriptive statistics and correlations between key variables*.*

	Steps	Wear time (h)	PM_10_	Temperature	Precipitation	Sunshine duration	Age	Runner	Male	Education	AP monitoring	Lockdown
Steps												
Wear time	0.181^a^											
PM_10_	−0.016	0.037^a^										
Temperature	0.122^a^	0.007	−0.264^a^									
Precipitation	−0.092^a^	−0.019	−0.148^a^	0.095^a^								
Sunshine duration	0.145^a^	0.018	0.017	0.572^a^	−0.254^a^							
Age	0.083^a^	0.116^a^	−0.012	−0.012	−0.024^a^	0.001						
Runner	0.301^a^	0.030^a^	−0.018	0.035^a^	−0.042^a^	0.018	−0.109^a^					
Male	0.066^a^	0.049^a^	0.032^a^	−0.056^a^	−0.008	−0.036^a^	−0.004	0.080^a^				
Education	−0.036^a^	0.003	0.002	−0.059^a^	−0.008	−0.040^a^	0.124^a^	0.030^a^	−0.072^a^			
AP monitoring	0.008	0.059^a^	−0.007	0.029^a^	−0.028^a^	0.028^a^	0.199^a^	0.066^a^	0.080^a^	0.126^a^		
Lockdown	0.015	0.034^a^	0.053^a^	−0.077^a^	−0.044^a^	0.044^a^	−0.024^a^	0.056^a^	−0.018	−0.016	−0.060^a^	
Mean (%)	13,934	15.41	21.72	12.08	2.62	4.85	38.78	58%	53%	45%	45%	2.6%
SD	8,140	2.57	13.38	7.66	6.10	4.14	12.28					
Min	67	0.02	1.57	−12.06	0	0	18					
Max	113,602	23.98	149.99	28.48	82.17	15.3	65					

^a^ Effect for which the 95% CI does not include 0.

### Multi‐level moderation model

Table 2 presents the final coefficients from the hypothesized multi‐level model predicting daily step count using a Bayesian estimator. A summary of model assumption checks (e.g., linearity, normality, homoscedasticity) is provided in Data S6.

**TABLE 2:** Coefficients predicting daily step count from multilevel moderation regression model with Bayesian estimator.

Predictor variable			95% CI	
	Estimate	*SD*_post_	Lower	Higher
Fixed effects for within‐person level (L1)				
Daily temperature	**0.066**	0.026	0.006	0.116
Daily precipitation	**−0.065**	0.013	−0.091	−0.039
Daily sunshine	**0.146**	0.025	0.096	0.194
Lockdown day (1: in lockdown)	−2.641	2.552	−7.713	2.285
Daily wear time	**0.537**	0.039	0.461	0.615
Fixed effects for between‐person (L2)				
Activity status (1: runner)	**4.819**	0.350	4.140	5.495
Gender (1: male)	0.522	0.340	−0.144	1.191
Education (1: university)	−0.672	0.342	−1.346	0.006
Age	**0.066**	0.014	0.038	0.093
Average PM_10_	−0.036	0.029	−0.093	0.020
Average precipitation	−0.302	0.169	−0.641	0.021
Average sunshine	**0.365**	0.075	0.216	0.511
Average wear time	**1.025**	0.175	0.689	1.373
Following AP status	**−0.751**	0.353	−1.440	−0.065
Average PM_10_ × following AP status	−0.046	0.043	−0.130	0.038
Intercepts				
Steps	**13.778**	0.172	13.433	14.111
Random slope for PM_10_	**−0.027**	0.010	−0.047	−0.006
Fixed effects for cross‐level interaction				
Daily PM_10_ × following AP status	−0.003	0.021	−0.044	0.038
Residual variances				
Residual variance in daily PM_10_ slope	**0.016**	0.004	0.008	0.025
Within‐person residual variance in steps	**37.213**	0.683	35.936	38.633
Between‐person residual variance in steps	**16.429**	1.103	14.411	18.752

*Note*: The effect for which the 95% CI does not include 0 is presented in bold. Estimate = median of posterior distribution; AP, air pollution; CI, credible interval; PM_10_, particulate matter 10; SD_post_, posterior standard deviation.

#### Within‐person effects

At the within‐person level, daily average PM₁₀ values were negatively related to daily step counts (*b* = −0.027; 95% CI = [−0.047, −0.006]). Specifically, on a day with higher PM₁₀ than average for the 2‐week burst, the participants recorded lower step counts. On average, a 1 SD increase in PM₁₀ was related to 361 steps less on that day.

As for the daily‐level covariates, daily air temperature (*b* = 0.066; 95% CI = [0.006, 0.116]) and daily sunshine duration (*b* = 0.146; 95% CI = [0.096, 0.194]) were positively related to daily step counts, whereas daily precipitation (*b* = −0.065; 95% CI = [−0.091, −0.039]) was negatively related to daily step counts. On average, a 1°C increase resulted in 66 more daily steps, one more hour of sunshine resulted in 146 more daily steps, and a 1 mm increase in precipitation resulted in 65 less daily steps. Daily Fitbit wear time was also positively associated with daily step counts (see Table 2).

#### Cross‐level moderation effects

Additionally, we evaluated the moderating effect of the self‐reported general AP monitoring tendency on the within‐person relationship between daily PM₁₀ and daily step counts. The cross‐level moderating effect was not credibly different from zero (*b* = −0.003; 95% CI = [−0.044, 0.038]), meaning that individuals who reported generally following AP status did not show a stronger reduction in steps on days with higher PM₁₀ levels compared to those who do not generally follow AP status.

#### Between‐person effects

At the between‐person level, person‐average AP and precipitation across the daily observations were not related to their average step counts. Only higher average sunshine hours were linked to higher average step counts. Individuals who report general AP monitoring tendency recorded lower average step counts. Nevertheless, this monitoring behavior did not moderate the relationship between average AP and step counts.

Additionally, being classified as a runner (as opposed to an inactive individual) was related to higher average step counts. Similarly, higher age was linked to higher average step counts. However, gender and education were not linked to average step counts. The respective statistical coefficients for the between‐person effects can be found in Table 2.

## DISCUSSION

The present study examined how daily background AP relates to daily fluctuations in step counts across a 2‐week period, while controlling for weather variables. It was further investigated whether general self‐reported AP monitoring tendency moderates the daily effect of AP on PA. The results showed that higher concentrations of PM₁₀ were associated with lower step counts on a given day. The moderating effect of general AP monitoring tendency on the within‐person relationship between AP and PA was not supported.

At the within‐person level, consistent with our hypothesis, daily PM₁₀ concentrations were negatively associated with daily step counts, even after adjusting for weather variables and wear time of the Fitbit device. This finding supports and extends previous research (An et al., 2018; Baillot et al., 2024; Yu et al., 2021) by showing that device‐based PA declines on days with relatively poorer air quality linked to participants' approximate locations.

Although the estimated within‐person coefficient appears small in absolute terms, specifically a reduction of 27 steps per 1‐unit increase in PM₁₀, it translates into a meaningful behavioral difference: in the present sample, a 1‐SD increase in PM₁₀ (≈13.4 μg/m^3^) was associated with approximately 360 fewer daily steps. In real‐world conditions, daily PM₁₀ concentrations in the region frequently fluctuate by tens of μg/m^3^, particularly during winter heating periods or temperature inversions; the average person‐specific daily PM₁₀ values in the present study ranged from 1.57 to 149.99. To contextualize these concentrations, the WHO 24‐h guideline for PM₁₀ is 45 μg/m^3^ (WHO, 2021), whereas the Czech national 24‐h emission limit declared to protect human health is 50 μg/m^3^ (permitted to be exceeded 35 times per year as per Act No. 201/2012 Coll. Act on Air Protection; Czech Republic, 2012). Thus, during episodes with substantially elevated pollution levels, reductions in step counts may reach the higher hundreds of steps per day—an amount of PA that has been linked to meaningful health effects (Ding et al., 2025; Yates et al., 2014).

On one the hand, the lower PA on days with relatively higher AP could be viewed as a desired behavioral adaptation preventing individuals from increased pollutant inhalation rate during PA (Tainio et al., 2021). Therefore, from a short‐term risk mitigation perspective, such behavioral adjustments might be seen as protective and beneficial in areas with relatively infrequent instances of high AP. On the other hand, regular PA is fundamental for long‐term health (Ding et al., 2025; WHO, 2020), and if individuals systematically reduce their movement because of frequent or persistent environmental threats, this may contribute to insufficient levels of PA, exacerbating risks for cardiovascular, metabolic, and mental health conditions (WHO, 2020). Moreover, some recent evidence indicates that the benefits of PA may not be outweighed by risks related to excess exposure to AP during PA (Chen et al., 2022; Tainio et al., 2021; Zhang et al., 2022), possibly except for certain high‐risk populations such as children (Tainio et al., 2021) or very high levels of pollution (Sun et al., 2023). Nevertheless, the specific operationalization of PA (e.g., self‐reported habitual PA vs. active commuting vs. accelerometer‐derived PA), differing regions of study (e.g., relatively low vs. high AP) as well as studied populations might contribute to these inconsistencies. Thus, this remains an emerging area of research with no clear consensus to date (Hahad et al., 2023; You et al., 2022).

However, at the between‐person level, average PM₁₀ exposure was not associated with differences in average step counts. This result should be interpreted cautiously because the between‐person PM₁₀ variable in this study does not fully reflect a stable, person‐specific exposure level. Because each participant contributed data from only one 14‐day burst, average PM₁₀ exposure was strongly influenced by the seasonal timing of assessment, given the substantial seasonal variation in AP levels (CHMI, 2021; see also Data S8 for monthly distributions of daily PM₁₀ values across the study period). Although PM₁₀ concentrations were generally higher during winter months, elevated levels were observed across multiple months rather than exclusively during winter. Therefore, the lack of a between‐person association should not be interpreted as evidence that long‐term exposure to higher AP is unrelated to PA; rather it suggests that the participants monitored during relatively more versus less polluted seasonal periods did not differ substantially in their overall daily step counts.

Furthermore, to examine the potential mechanisms underlying the negative effect of AP on PA, we examined whether AP monitoring tendency moderated this within‐person relationship as conceptually informed by the Protection Motivation Theory (Rogers, 1983). Nevertheless, contrary to our expectations that monitoring AP may serve as a proxy for heightened threat appraisal and thus be associated with reduced PA on high‐pollution days as well as some previous research (e.g., Saberian et al., 2017; Wen et al., 2009), individuals who reported generally following AP status did not show stronger reductions in step counts on days with higher PM₁₀ than average.

Importantly, this null finding should be interpreted with caution. The present measure of AP monitoring tendency was based on a single baseline self‐report item and does not capture whether the participants actually engaged in day‐level monitoring behavior. However, it is precisely such momentary monitoring and information use that would be theoretically relevant for triggering threat appraisal and subsequent behavioral adjustment within the PMT framework. Therefore, the absence of a moderating effect may reflect limitations in the measurement of the moderator rather than the absence of a PMT‐consistent process. Accordingly, the present findings should not be interpreted as evidence against the PMT hypothesis per se but rather highlight the need for more fine‐grained, time‐sensitive assessments of monitoring behavior in future research. This could include more direct behavioral indicators of pollution‐related decision‐making, such as app usage logs from air‐quality monitoring tools, geolocation‐based avoidance of high‐pollution areas, or EMAs capturing real‐time decisions in response to air‐quality information (e.g., Park et al., 2023).

Beyond measurement limitations, one possible explanation for the absence of a moderating effect is that individuals aware of AP might have adapted their behavior to indoor PA options rather than reducing activity altogether (Wangsan et al., 2024). In the present study, we evaluated daily step counts as an indicator of overall levels of PA, encompassing the typical outdoor activities such as walking and running. Nevertheless, our data did not allow us to reliably determine the proportion of outdoor versus indoor step counts and thus account for such adaptation behaviors. Interestingly, those who reported monitoring AP also had lower average step counts across the 14‐day period. While speculative, this could suggest that AP monitoring behavior may coincide with risk‐averse or avoidant behavior patterns—particularly in industrial areas where opportunities for safe outdoor activity are limited.

Importantly, the absence of a moderating effect of general monitoring behavior tendency does not preclude the role of other pathways linking AP with reduced PA. Physiological responses to acute pollution exposure (e.g., respiratory discomfort, headaches, fatigue) and pollution‐induced impairments in decision‐making may directly discourage activity, independent of conscious threat appraisal (e.g., Aguilar‐Gomez et al., 2022; Cutrufello et al., 2011; Dominski et al., 2021; Manisalidis et al., 2020). Additionally, sensory cues, such as visible smog or unpleasant odor, might trigger avoidance of outdoor activity even in the absence of explicit information monitoring. Thus, future studies should focus on evaluating these potential pathways and could try to disentangle the relative influences of information, sensation, and bodily feedback on daily PA levels in air polluted environments.

### Implications

Importantly, the present findings have implications for promoting PA in real‐world settings. This study highlights how short‐term AP exposure is associated with daily movement patterns, reinforcing the need for responsive public health strategies that account for such fluctuating conditions. Nevertheless, AP should not be considered in isolation, but rather as one potentially useful component within a broader set of dynamic influences affecting PA. Combining environmental indicators, such as AP and weather with additional behavioral, psychological, and physiological sources, may contribute to more precise multidimensional early‐warning signals of upcoming reductions in PA (Baretta et al., 2025). This multidimensional approach could be especially useful for the development of just‐in‐time adaptive interventions that would include context‐sensitive health messaging, for example, based on current AP levels and weather (Wunsch et al., 2022). Furthermore, the present results underscore the need for investment in infrastructure that facilitates safe and accessible activity in adverse conditions, including well‐ventilated indoor exercise spaces and green and blue infrastructure (Kumar et al., 2024), as well as the integration of environmental data into digital health tools, such as apps that adjust suggestions based on forecasted AP (Strusi et al., 2022). These approaches can help individuals maintain regular activity despite barriers in their immediate environment, particularly in regions more vulnerable to AP. Ultimately, recognizing and accommodating the dynamic nature of environmental exposures is essential for designing equitable and sustainable PA promotion efforts.

### Strengths and limitations

The strength of the present study lies in utilizing 14 days of intensive longitudinal data on device‐based PA and passively sensed location proxies, matched with AP and weather data from the nearest background stations in a sample of 693 participants living in an industrial region. Thus, it provides an ecologically valid assessment of real‐life behavior, capturing the day‐to‐day dynamics between AP and PA while controlling for relevant weather variables.

With respect to the environmental exposure data, the present study extends the previous studies that used a single AP station for the entire sample (e.g., Alahmari et al., 2015) by linking smartphone‐derived location proxies to the nearest monitoring station at the level of an individual location proxy reading. Although daily AP exposures were derived from nearest representative background stations rather than direct personal exposure measurements (e.g., from wearable air‐quality monitors or exposure modeling based on continuous location data), this approach provided a more spatially sensitive estimate of background AP exposure than relying on a single fixed station for the entire sample or estimating AP only for participants' residential addresses.

Moreover, the study was conducted in the Moravian–Silesian Region, a highly polluted industrial area that has been underrepresented in PA and environmental exposure research (e.g., Kim et al., 2021; Tainio et al., 2021). As previous reviews have highlighted the over‐representation of studies from China and the United States and the need for evidence from diverse settings, the current work broadens geographic and environmental coverage in this literature.

Several limitations should be acknowledged when interpreting our findings. This study is based on a large intensive longitudinal dataset (14 days, 693 participants), but longer time‐series would have ensured better examination of the within‐person association between environmental exposure variables and PA. Although the broader 4HAIE project included repeated monitoring bursts across 1 year, the present analyses focused only on the first 14‐day burst per participant because of data quality considerations (see  Present study data). As a result, each participant contributed data from only one period of the year, which may have influenced the variability of within‐person PM₁₀ exposure and the strength of the observed association. At the same time, the participants' first burst occurred between April 2019 and September 2021, meaning that PM₁₀–PA associations were examined across a wide range of seasonal and environmental conditions at the sample level (see Figure S8 for monthly distributions of daily PM₁₀ values across the study period). In the present study, latent cluster mean centering helped separate within‐person deviations in PM₁₀ from stable between‐person seasonal differences, and the inclusion of daily weather covariates (temperature, sunshine, precipitation) further reduced potential seasonal confounding. Nevertheless, future studies would benefit from analyses spanning multiple seasons within the same individuals to better capture seasonal variation in both AP exposure and PA.

Environmental exposure assessment also presents several related limitations. The participant location was estimated using smartphone‐based location proxies recorded only when participants interacted with the study app, rather than through continuous location tracking or direct personal exposure monitoring. Consequently, these data represent snapshots of daily whereabouts rather than continuous mobility patterns. In addition, our analysis focused solely on daily PM₁₀ values derived from representative background monitoring stations. While this approach provided stable and spatially consistent estimates of background AP exposure, it does not fully capture direct personal exposure or highly localized pollution sources such as traffic or industrial hotspots. Similarly, relying on PM₁₀ alone may not reflect the full range of relevant pollutants and may underestimate exposure during periods when other pollutants, such as ozone, are more prominent, particularly during summer months (Bernard et al., 2021; Meleux et al., 2007). Our reliance on background stations rather than hotspot monitoring sites was intentional, as hotspot measurements are highly localized (typically within tens of meters) and participant proximity to these specific sites could not be reliably determined within the study design. Background stations therefore provided a more stable estimate of general ambient exposure, whereas PM₁₀ offered the broadest and most consistent monitoring coverage within the Czech air‐quality network (see Location‐based data). However, these context‐specific analytic decisions may reduce sensitivity to short‐term pollution peaks in specific microenvironments, such as traffic corridors or industrial zones, and may limit the broader generalizability of findings to other pollutants, hotspot exposure, or settings with different monitoring systems.

In addition, although data on official PM₁₀ air‐pollution alerts (smog situations) were available, they were not included in the present analyses because such alerts were rare during the study period and reflect only extreme pollution episodes rather than typical day‐to‐day exposure variability. As a result, they were less informative for understanding habitual PA patterns. Nevertheless, future research could examine both dynamic fluctuations in AP and formal alert periods, while incorporating individual‐level exposure data that better capture the variability of microenvironments throughout the day, including indoor pollution.

To address some of these limitations, future research should incorporate continuous passive location sensing and wearable personal air‐quality monitors capable of capturing fine‐scale spatial and temporal variability, including indoor exposure and short‐lived pollution peaks. Although regulatory monitoring stations provide certified and methodologically stable air‐quality data, portable sensors can complement these data by offering denser spatial coverage and better detection of local microenvironmental exposures relevant to daily PA. Because such devices may show lower precision and require calibration against reference instruments (Nieckarz & Zoladz, 2020), future studies should carefully balance ecological validity and measurement reliability according to the specific research question.

A significant limitation concerns the AP monitoring tendency variable, which was measured using a single yes/no item at baseline. This measure could not capture the frequency, context, or day‐level variability of monitoring behavior, nor whether participants actually checked air quality information on high‐pollution days. As a result, it may not adequately reflect the actual monitoring behaviors relevant for day‐to‐day behavioral adaptation. Future research should incorporate more granular and dynamic assessments of AP monitoring behavior to distinguish between‐person differences in general monitoring tendency from within‐person day‐level fluctuations in actual monitoring behavior.

Finally, the operationalization of PA, particularly its temporal and contextual resolution, warrants consideration as well. We analyzed total daily step counts, which do not allow for fine‐grained assessment of when or where PA occurred. As a result, we were unable to determine whether activity coincided with periods and areas of particularly high AP, or whether it was of moderate or vigorous intensity (when inhalation rates increase). Thus, future research may benefit from analyzing the PA data at a more granular level as well as from incorporating passive location sensing continuously throughout the day.

### Conclusion

This study provides novel insights into how daily environmental conditions, specifically background AP, shape PA behavior in real‐world contexts. Using device‐based measures of step count and environmental exposures in a central European industrial region, we found that individuals were less active on days with higher background AP while controlling for the effect of weather. These within‐person associations highlight the importance of short‐term environmental fluctuations in influencing health behavior and reinforce the value of intensive longitudinal data. Contrary to our expectations, general AP monitoring tendency did not moderate the daily relationship between AP and PA. This suggests that the observed behavioral changes may occur regardless of self‐reported awareness of air quality status and that other potential mechanisms need to be explored.

These findings have particular relevance in light of climate change, which is expected to amplify the variability and extremity of daily environmental conditions, posing new challenges to maintaining regular PA. Public health strategies and future interventions should leverage personalized, context‐aware tools and infrastructures that enable individuals to adapt their PA routines to increasingly variable environmental conditions without sacrificing overall movement. Ultimately, integrating environmental dynamics into health behavior research and promotion is crucial for developing equitable and sustainable strategies that meet the needs of diverse populations, including those living in highly polluted or socioeconomically disadvantaged areas, in a changing climate.

## CONFLICT OF INTEREST STATEMENT

There are no conflicts of interest associated with this manuscript or its co‐authors.

## ETHICS STATEMENT

The study was conducted in accordance with the Declaration of Helsinki and was approved by an Institutional Review Board/Ethics committee. See details under Methods.

## Supporting information


**Data S1.** **Model equations.** Full specification of the two‐level multilevel model, including within‐person and between‐person equations, random effects, and cross‐level interactions.
**Data S2. Mplus code.** Simplified Mplus syntax used for estimating the multilevel Bayesian model.
**Data S3. Bayesian estimation details.** Description of the Bayesian estimation procedure, prior distributions, Markov Chain Monte Carlo settings, convergence assessment, and estimation criteria.
**Data S4. Simulation‐based power analysis.** Monte Carlo simulation code and procedures used to evaluate statistical power.
**Data S5. Examination of nonlinear effects of PM₁₀.** Generalized additive model analyses assessing potential nonlinear associations between daily PM₁₀ concentrations and daily step counts, including supplementary results and figures.
**Data S6. Model assumptions check.** Diagnostic analyses evaluating linearity, homoscedasticity, normality of residuals, and random‐effects distributions, with accompanying diagnostic plots.
**Data S7. Location‐based data – technical appendix.** Detailed description of the location‐proxy methodology, linkage of participant locations to air‐quality monitoring stations, exposure assignment procedures, privacy safeguards, and data‐quality filtering criteria.
**Data S8. Monthly distributions of daily PM₁₀ concentrations across all study background stations.** Supplementary figure presenting monthly distributions of PM₁₀ concentrations during the study period.

## Data Availability

The data used in this study are available upon request as per data sharing policy of the Healthy Aging in Industrial Environment HAIE project at https://haie-lerco.cz/data/.

## References

[aphw70186-bib-0001] Aguilar‐Gomez, S. , Dwyer, H. , Graff Zivin, J. , & Neidell, M. (2022). This is air: The “nonhealth” effects of air pollution. Annual Review of Resource Economics, 14 (1), 403–425. 10.1146/annurev-resource-111820-021816

[aphw70186-bib-0002] Alahmari, A. D. , Mackay, A. J. , Patel, A. R. C. , Kowlessar, B. S. , Singh, R. , Brill, S. E. , Allinson, J. P. , Wedzicha, J. A. , & Donaldson, G. C. (2015). Influence of weather and atmospheric pollution on physical activity in patients with COPD. Respiratory Research, 16 (1), 1–015. 10.1186/s12931-015-0229-z 26071400 PMC4470337

[aphw70186-bib-0003] An, R. , Shen, J. , Ying, B. , Tainio, M. , Andersen, Z. J. , & de Nazelle, A. (2019). Impact of ambient air pollution on physical activity and sedentary behavior in China: A systematic review. Environmental Research, 176 (January), 108545. 10.1016/j.envres.2019.108545 31280030

[aphw70186-bib-0004] An, R. , Zhang, S. , Ji, M. , & Guan, C. (2018). Impact of ambient air pollution on physical activity among adults: A systematic review and meta‐analysis. Perspectives in Public Health, 138 (2), 111–121. 10.1177/1757913917726567 28829249

[aphw70186-bib-0005] Arend, M. G. , & Schäfer, T. (2019). Statistical power in two‐level models: A tutorial based on Monte Carlo simulation. Psychological Methods, 24 (1), 1–19. 10.1037/met0000195 30265048

[aphw70186-bib-0006] Asparouhov, T. , & Muthén, B. (2010). Bayesian analysis of latent variable models using Mplus *(Technical Report)*. Version 5. Retrieved from https://www.statmodel.com/download/BayesAdvantages18.pdf.

[aphw70186-bib-0007] Asparouhov, T. , & Muthén, B. (2019). Latent variable centering of predictors and mediators in multilevel and time‐series models. Structural Equation Modeling: A Multidisciplinary Journal, 26 (1), 119–142. 10.1080/10705511.2018.1511375

[aphw70186-bib-0096] Baillot, A. , Bernard, P. , Eddine, J. N. , Thomas, J. G. , Schumacher, L. M. , Papasavas, P. K. , Vithiananthan, S. , Jones, D. , & Bond, D. S. (2024). Associations of weather and air pollution with objective physical activity and sedentary time before and after bariatric surgery: A secondary analysis of a prospective cohort study. Environmental Research Communications, 6 (8), 085003. 10.1088/2515-7620/ad64b2 39469319 PMC11514705

[aphw70186-bib-0009] Bandura, A. (1986). Social foundations of thought and action: A social cognitive theory. Prentice Hall.

[aphw70186-bib-0010] Baretta, D. , Koch, S. , Buekers, J. , Garcia‐Aymerich, J. , Knapova, L. , Elavsky, S. , Godino, J. , Olthof, M. , Lichtwarck‐Aschoff, A. , den Hartigh, R. , & Chevance, G. (2025). Predicting recovery after stressors using step count data derived from activity monitors. npj Digital Medicine, 8 (1), 606. 10.1038/s41746-025-01998-0 41068331 PMC12511605

[aphw70186-bib-0011] Bernard, P. , Chevance, G. , Kingsbury, C. , Baillot, A. , Romain, A. J. , Molinier, V. , Gadais, T. , & Dancause, K. N. (2021). Climate change, physical activity and sport: A systematic review. Sports Medicine, 51 (5), 1041–1059. 10.1007/s40279-021-01439-4 33689139

[aphw70186-bib-0012] Chan, C. B. , Ryan, D. A. J. , & Tudor‐Locke, C. (2006). Relationship between objective measures of physical activity and weather: A longitudinal study. International Journal of Behavioral Nutrition and Physical Activity, 3, 1–21. 10.1186/1479-5868-3-21 16893452 PMC1557535

[aphw70186-bib-0013] Chen, L. , Cai, M. , Li, H. , Wang, X. , Tian, F. , Wu, Y. , Zhang, Z. , & Lin, H. (2022). Risk/benefit tradeoff of habitual physical activity and air pollution on chronic pulmonary obstructive disease: Findings from a large prospective cohort study. BMC Medicine, 20 (1), 1–12. 10.1186/s12916-022-02274-8 35220974 PMC8883705

[aphw70186-bib-0014] Cheng, J. , Wu, Y. , Wang, X. , & Yu, H. (2024). Objectively measured the impact of ambient air pollution on physical activity for older adults. BMC Public Health, 24 (1), 821. 10.1186/s12889-024-18279-2 38491436 PMC10941607

[aphw70186-bib-0015] Cho, Y. W. , Chow, S. M. , Marini, C. M. , & Martire, L. M. (2024). Multilevel latent differential structural equation model with short time series and time‐varying covariates: A comparison of frequentist and Bayesian estimators. Multivariate Behavioral Research, 59 (5), 934–956. 10.1080/00273171.2024.2347959 38821115 PMC11424268

[aphw70186-bib-0016] Cipryan, L. , Kutac, P. , Dostal, T. , Zimmermann, M. , Krajcigr, M. , Jandackova, V. , Sram, R. , Jandacka, D. , & Hofmann, P. (2020). Regular running in an air‐polluted environment: Physiological and anthropometric protocol for a prospective cohort study (Healthy aging in industrial environment study – Program 4). BMJ Open, 10 (12), 1–040529. 10.1136/bmjopen-2020-040529 PMC7733192 33303450

[aphw70186-bib-0017] Clayton, C. J. , Turnock, S. T. , Marsh, D. R. , Graham, A. M. , Reddington, C. L. , Vohra, K. , & McQuaid, J. B. (2025). Reducing inequities in the future air pollution health burden over Europe. Earth's Future, 13 (5), e2024EF005404. 10.1029/2024EF005404

[aphw70186-bib-0018] Cutrufello, P. T. , Rundell, K. W. , Smoliga, J. M. , & Stylianides, G. A. (2011). Inhaled whole exhaust and its effect on exercise performance and vascular function. Inhalation Toxicology, 23 (11), 658–667. 10.3109/08958378.2011.604106 21867399

[aphw70186-bib-0019] Czech Hydrometeorological Institute . (2021). Air pollution in the Czech Republic in 2021 (Vol. 2021). Accessed November 28, 2025. https://intranet.chmi.cz/files/portal/docs/uoco/isko/grafroc/21groc/gr21cz/Obsah_CZ.html.

[aphw70186-bib-0020] Czech Hydrometeorological Institute . (2025). Open data on air quality, hydrology and meteorology [Data set]. https://opendata.chmi.cz/.

[aphw70186-bib-0022] Czech Republic . (2012). Act No. 201/2012 Coll., on air protection. Collection of Laws of the Czech Republic.

[aphw70186-bib-0023] D'Antoni, D. , Smith, L. , Auyeung, V. , & Weinman, J. (2017). Psychosocial and demographic predictors of adherence and non‐adherence to health advice accompanying air quality warning systems: A systematic review. Environmental Health: A Global Access Science Source, 16 (1), 1–4. 10.1186/s12940-017-0307-4 28938911 PMC5610416

[aphw70186-bib-0024] Dauchet, L. , Hulo, S. , Cherot‐Kornobis, N. , Matran, R. , Amouyel, P. , Edmé, J. L. , & Giovannelli, J. (2018). Short‐term exposure to air pollution: Associations with lung function and inflammatory markers in non‐smoking, healthy adults. Environment International, 121 (Pt 1), 610–619. 10.1016/j.envint.2018.09.036 30312964

[aphw70186-bib-0025] Deguen, S. , Ségala, C. , Pédrono, G. , & Mesbah, M. (2012). A new air quality perception scale for global assessment of air pollution health effects. Risk Analysis: An Official Publication of the Society for Risk Analysis, 32 (12), 2043–6924. 10.1111/j.1539-6924.2012.01862.x 22852801

[aphw70186-bib-0026] Ding, D. , Nguyen, B. , Nau, T. , Luo, M. , Del Pozo Cruz, B. , Dempsey, P. C. , Munn, Z. , Jefferis, B. J. , Sherrington, C. , Calleja, E. A. , Hau Chong, K. , Davis, R. , Francois, M. E. , Tiedemann, A. , Biddle, S. J. H. , Okely, A. , Bauman, A. , Ekelund, U. , Clare, P. , & Owen, K. (2025). Daily steps and health outcomes in adults: A systematic review and dose‐response meta‐analysis. The Lancet. Public Health, 10 (8), e668–e681. 10.1016/S2468-2667(25)00164-1 40713949

[aphw70186-bib-0027] Dominski, F. H. , Lorenzetti Branco, J. H. , Buonanno, G. , Stabile, L. , Gameiro da Silva, M. , & Andrade, A. (2021). Effects of air pollution on health: A mapping review of systematic reviews and meta‐analyses. Environmental Research, 201 (April), 111487. 10.1016/j.envres.2021.111487 34116013

[aphw70186-bib-0028] Ekelund, U. , Tarp, J. , Steene‐Johannessen, J. , Hansen, B. H. , Jefferis, B. , Fagerland, M. W. , Whincup, P. , Diaz, K. M. , Hooker, S. P. , Chernofsky, A. , Larson, M. G. , Spartano, N. , Vasan, R. S. , Dohrn, I. M. , Hagströmer, M. , Edwardson, C. , Yates, T. , Shiroma, E. , Anderssen, S. A. , & Lee, I. M. (2019). Dose‐response associations between accelerometry measured physical activity and sedentary time and all cause mortality: Systematic review and harmonised meta‐analysis. BMJ, 366, 1–10. 10.1136/bmj.l4570 PMC6699591 31434697

[aphw70186-bib-0029] Elavsky, S. , Jandačková, V. , Knapová, L. , Vašendová, V. , Sebera, M. , Kaštovská, B. , Blaschová, D. , Kühnová, J. , Cimler, R. , Vilímek, D. , Bosek, T. , Koenig, J. , & Jandačka, D. (2021). Physical activity in an air‐polluted environment: Behavioral, psychological and neuroimaging protocol for a prospective cohort study (Healthy aging in industrial environment study – Program 4). BMC Public Health, 21 (1), 126. 10.1186/s12889-021-10166-4 33435943 PMC7801866

[aphw70186-bib-0030] European Environment Agency . (2022). Air pollution and health [Zero pollution monitoring assessment]. EEA. https://www.eea.europa.eu/publications/zero‐pollution/health/air‐pollution

[aphw70186-bib-0031] European Environment Agency . (2024). Harm to human health from air pollution in Europe: Burden of disease status, 2024 (Briefing No. 21/2024). https://www.eea.europa.eu/en/analysis/publications/harm‐to‐human‐health‐from‐air‐pollution‐2024.

[aphw70186-bib-0032] Feehan, L. M. , Geldman, J. , Sayre, E. C. , Park, C. , Ezzat, A. M. , Yoo, J. Y. , Hamilton, C. B. , & Li, L. C. (2018). Accuracy of Fitbit devices: Systematic review and narrative syntheses of quantitative data. JMIR mHealth and uHealth, 6 (8), e10527. 10.2196/10527 30093371 PMC6107736

[aphw70186-bib-0033] Ferguson, T. , Curtis, R. , Fraysse, F. , Olds, T. , Dumuid, D. , Brown, W. , Esterman, A. , & Maher, C. (2023). Weather associations with physical activity, sedentary behaviour and sleep patterns of Australian adults: a longitudinal study with implications for climate change. International Journal of Behavioral Nutrition and Physical Activity, 20 (1), 30. 10.1186/s12966-023-01414-4 36918954 PMC10012316

[aphw70186-bib-0034] Fuller, R. , Landrigan, P. J. , Balakrishnan, K. , Bathan, G. , Bose‐O'Reilly, S. , Brauer, M. , Caravanos, J. , Chiles, T. , Cohen, A. , Corra, L. , Cropper, M. , Ferraro, G. , Hanna, J. , Hanrahan, D. , Hu, H. , Hunter, D. , Janata, G. , Kupka, R. , Lanphear, B. , … Yan, C. (2022). Pollution and health: a progress update. The Lancet Planetary Health, 6 (6), e535–e547. 10.1016/S2542-5196(22)00090-0 35594895 PMC11995256

[aphw70186-bib-0035] Guthold, R. , Stevens, G. A. , Riley, L. M. , & Bull, F. C. (2018). Worldwide trends in insufficient physical activity from 2001 to 2016: A pooled analysis of 358 population‐based surveys with 1·9 million participants. The Lancet Global Health, 6 (10), e1077–e1086. 10.1016/S2214-109X(18)30357-7 30193830

[aphw70186-bib-0036] Hahad, O. , Daiber, A. , & Münzel, T. (2023). Physical activity in polluted air: an urgent call to study the health risks. The Lancet Planetary Health, 7 (4), e266–e267. 10.1016/S2542-5196(23)00055-4 37019566

[aphw70186-bib-0037] HealthReact . (n.d.). HealthReact – Advanced mHealth platform for research and healthcare. Retrieved December 1, 2025, from https://www.healthreact.eu/.

[aphw70186-bib-0038] Henriksen, A. , Haugen Mikalsen, M. , Woldaregay, A. Z. , Muzny, M. , Hartvigsen, G. , Hopstock, L. A. , & Grimsgaard, S. (2018). Using fitness trackers and smartwatches to measure physical activity in research: Analysis of consumer wrist‐worn wearables. Journal of Medical Internet Research, 20 (3), e110. 10.2196/jmir.9157 29567635 PMC5887043

[aphw70186-bib-0039] Ho, J. Y. , Goggins, W. B. , Mo, P. K. H. , & Chan, E. Y. Y. (2022). The effect of temperature on physical activity: an aggregated timeseries analysis of smartphone users in five major Chinese cities. International Journal of Behavioral Nutrition and Physical Activity, 19 (1), 1–15. 10.1186/s12966-022-01285-1 35701809 PMC9195465

[aphw70186-bib-0040] Jandacka, D. , Uchytil, J. , Zahradnik, D. , Farana, R. , Vilimek, D. , Skypala, J. , Urbaczka, J. , Plesek, J. , Motyka, A. , Blaschova, D. , Beinhauerova, G. , Rygelova, M. , Brtva, P. , Balazova, K. , Horka, V. , Malus, J. , Silvernail, J. F. , Irwin, G. , Nieminen, M. T. , … Hamill, J. (2020). Running and physical activity in an air‐polluted environment: The biomechanical and musculoskeletal protocol for a prospective cohort study 4HAIE (Healthy aging in industrial environment – Program 4). International Journal of Environmental Research and Public Health, 17 (23), 9142. 10.3390/ijerph17239142 33297585 PMC7730319

[aphw70186-bib-0041] Josa‐Culleré, A. , Basagaña, X. , Koch, S. , Arbillaga‐Etxarri, A. , Balcells, E. , Bosch de Basea, M. , Celorrio, N. , Foraster, M. , Rodriguez‐Roisin, R. , Marin, A. , Peralta, G. P. , Rodríguez‐Chiaradia, D. A. , Simonet, P. , Torán‐Monserrat, P. , Vall‐Casas, P. , & Garcia‐Aymerich, J. (2024). Short‐term effects of air pollution and weather on physical activity in patients with chronic obstructive pulmonary disease (COPD). Environmental Research, 247, 118195. 10.1016/j.envres.2024.118195 38237751

[aphw70186-bib-0042] Kim, Y. B. , McCurdy, A. P. , Lamboglia, C. G. , Hakimi, S. , Kuzik, N. , Lee, E. Y. , Lindeman, C. , Sivak, A. , & Spence, J. C. (2021). Ambient air pollution and movement behaviours: A scoping review. Health & Place, 72 (December 2020), 102676. 10.1016/j.healthplace.2021.102676 34700061

[aphw70186-bib-0043] Klenk, J. , Büchele, G. , Rapp, K. , Frankev, S. , Peter, R. , Nikolaus, T. , Denkinger, M. , Nagel, G. , Weinmayr, G. , Herbolsheimer, F. , Ludolph, A. C. , von Arnim, C. A. F. , Scharffetter‐Kochanek, K. , Geiger, H. , Steinacker, J. M. , Böhm, B. O. , Kirchheiner, J. , Koenig, W. , Schumann, C. , … Ludolph, L. (2012). Walking on sunshine: effect of weather conditions on physical activity in older people: Figure 1. Journal of Epidemiology and Community Health, 66 (5), 474–476. 10.1136/jech.2010.128090 21325149

[aphw70186-bib-0044] Kocot, K. , & Zejda, J. E. (2021). Acute cardiorespiratory response to ambient air pollution exposure during short‐term physical exercise in young males. Environmental Research, 195, 110746. 10.1016/j.envres.2021.110746 33484723

[aphw70186-bib-0045] Kruschke, J. K. (2021). Bayesian analysis reporting guidelines. Nature Human Behaviour, 5 (10), 1282–1291. 10.1038/s41562-021-01177-7 PMC8526359 34400814

[aphw70186-bib-0046] Kumar, P. , Debele, S. E. , Khalili, S. , Halios, C. H. , Sahani, J. , Aghamohammadi, N. , Andrade, M. d. F. , Athanassiadou, M. , Bhui, K. , Calvillo, N. , Cao, S. J. , Coulon, F. , Edmondson, J. L. , Fletcher, D. , Dias de Freitas, E. , Guo, H. , Hort, M. C. , Katti, M. , Kjeldsen, T. R. , … Jones, L. (2024). Urban heat mitigation by green and blue infrastructure: Drivers, effectiveness, and future needs. The Innovation, 5 (2), 100588. 10.1016/j.xinn.2024.100588 38440259 PMC10909648

[aphw70186-bib-0047] Luszczynska, A. , & Schwarzer, R. (2005). Social cognitive theory. In M. Conner & P. Norman (Eds.), Predicting health behaviour (2nd ed.) (pp. 127–169). Open University Press.

[aphw70186-bib-0048] Machaczka, O. , Jiřík, V. , Janulková, T. , Michalík, J. , Siemiatkowski, G. , Osrodka, L. , Krajny, E. , & Topinka, J. (2023). Comparisons of lifetime exposures between differently polluted areas and years of life lost due to all‐cause mortality attributable to air pollution. Environmental Sciences Europe, 35 (1), 73. 10.1186/s12302-023-00778-5

[aphw70186-bib-0049] Manisalidis, I. , Stavropoulou, E. , Stavropoulou, A. , & Bezirtzoglou, E. (2020). Environmental and health impacts of air pollution: A review. Frontiers in Public Health, 8, 14. 10.3389/fpubh.2020.00014 32154200 PMC7044178

[aphw70186-bib-0050] Marquez, D. X. , Aguinãga, S. , Vásquez, P. M. , Conroy, D. E. , Erickson, K. I. , Hillman, C. , Stillman, C. M. , Ballard, R. M. , Sheppard, B. B. , Petruzzello, S. J. , King, A. C. , & Powell, K. E. (2020). A systematic review of physical activity and quality of life and well‐being. Translational Behavioral Medicine, 10 (5), 1098–1109. 10.1093/tbm/ibz198 33044541 PMC7752999

[aphw70186-bib-0051] McCormack, G. R. , & Shiell, A. (2011). In search of causality: A systematic review of the relationship between the built environment and physical activity among adults. International Journal of Behavioral Nutrition and Physical Activity, 8 (125), 125. 10.1186/1479-5868-8-125 22077952 PMC3306205

[aphw70186-bib-0052] McDowell, C. P. , Dishman, R. K. , Gordon, B. R. , & Herring, M. P. (2019). Physical activity and anxiety: A systematic review and meta‐analysis of prospective cohort studies. American Journal of Preventive Medicine, 57 (4), 545–556. 10.1016/j.amepre.2019.05.012 31542132

[aphw70186-bib-0053] Meleux, F. , Solmon, F. , & Giorgi, F. (2007). Increase in summer European ozone amounts due to climate change. Atmospheric Environment, 41 (35), 7577–7587. 10.1016/j.atmosenv.2007.05.048

[aphw70186-bib-0054] Michie, S. , van Stralen, M. M. , & West, R. (2011). The behaviour change wheel: A new method for characterising and designing behaviour change interventions. Implementation Science: IS, 6 (42), 42. 10.1186/1748-5908-6-42 21513547 PMC3096582

[aphw70186-bib-0055] Muthén, B. , & Asparouhov, T. (2012). Bayesian structural equation modeling: A more flexible representation of substantive theory. Psychological Methods, 17 (3), 313–335. 10.1037/a0026802 22962886

[aphw70186-bib-0056] Muthén, L. K. , & Muthén, B. O. (2017). Mplus user's guide: Statistical analysis with latent variables (8th ed.). Muthén & Muthén.

[aphw70186-bib-0057] Nieckarz, Z. , & Zoladz, J. A. (2020). Low‐cost air pollution monitoring system—An opportunity for reducing the health risk associated with physical activity in polluted air. PeerJ, 8, e10041. 10.7717/peerj.10041 33062442 PMC7533060

[aphw70186-bib-0058] Noël, C. , Van Landschoot, L. , Vanroelen, C. , & Gadeyne, S. (2022). The public's perceptions of air pollution. What's in a name? Environmental Health Insights, 16, 11786302221123563. 10.1177/11786302221123563 36161068 PMC9500264

[aphw70186-bib-0059] Oravecz, Z. , Tuerlinckx, F. , & Vandekerckhove, J. (2011). A hierarchical latent stochastic differential equation model for affective dynamics. Psychological Methods, 16 (4), 468–490. 10.1037/a0024375 21823796

[aphw70186-bib-0060] Paluch, A. E. , Bajpai, S. , Bassett, D. R. , Carnethon, M. R. , Ekelund, U. , Evenson, K. R. , Galuska, D. A. , Jefferis, B. J. , Kraus, W. E. , Lee, I. M. , Matthews, C. E. , Omura, J. D. , Patel, A. V. , Pieper, C. F. , Rees‐Punia, E. , Dallmeier, D. , Klenk, J. , Whincup, P. H. , Dooley, E. E. , … Steps for Health Collaborative . (2022). Daily steps and all‐cause mortality: a meta‐analysis of 15 international cohorts. The Lancet. Public Health, 7 (3), e219–e228. 10.1016/S2468-2667(21)00302-9 35247352 PMC9289978

[aphw70186-bib-0061] Park, Y. M. , Chavez, D. , Sousan, S. , Figueroa‐Bernal, N. , Alvarez, J. R. , & Rocha‐Peralta, J. (2023). Personal exposure monitoring using GPS‐enabled portable air pollution sensors: A strategy to promote citizen awareness and behavioral changes regarding indoor and outdoor air pollution. Journal of Exposure Science & Environmental Epidemiology, 33 (3), 347–357. 10.1038/s41370-022-00515-9 36513791 PMC10238623

[aphw70186-bib-0062] Pearce, M. , Garcia, L. , Abbas, A. , Strain, T. , Schuch, F. B. , Golubic, R. , Kelly, P. , Khan, S. , Utukuri, M. , Laird, Y. , Mok, A. , Smith, A. , Tainio, M. , Brage, S. , & Woodcock, J. (2022). Association between physical activity and risk of depression: A systematic review and meta‐analysis. JAMA Psychiatry, 79 (6), 550–559. 10.1001/jamapsychiatry.2022.0609 35416941 PMC9008579

[aphw70186-bib-0064] Reichert, M. , Giurgiu, M. , Koch, E. , Wieland, L. M. , Lautenbach, S. , Neubauer, A. B. , von Haaren‐Mack, B. , Schilling, R. , Timm, I. , Notthoff, N. , Marzi, I. , Hill, H. , Brüβler, S. , Eckert, T. , Fiedler, J. , Burchartz, A. , Anedda, B. , Wunsch, K. , Gerber, M. , … Liao, Y. (2020). Ambulatory assessment for physical activity research: state of the science, best practices and future directions. Psychology of Sport and Exercise, 50, 101742. 10.1016/j.psychsport.2020.101742 32831643 PMC7430559

[aphw70186-bib-0065] Richardson, E. A. , Pearce, J. , Tunstall, H. , Mitchell, R. , & Shortt, N. K. (2013). Particulate air pollution and health inequalities: A Europe‐wide ecological analysis. International Journal of Health Geographics, 12. 10.1186/1476-072X-12-34 PMC3720269 23866049

[aphw70186-bib-0066] Rogers, R. W. (1983). Cognitive and physiological processes in fear appeals and attitude change: A revised theory of protection motivation. In J. Cacioppo & R. Petty (Eds.), Social Psychophysiology (pp. 153–177). Guilford Press.

[aphw70186-bib-0067] Rosenstock, I. M. (1990). The health belief model: Explaining health behavior through expectancies. In K. Glanz , F. M. Lewis , & B. K. Rimer (Eds.), Health behavior and health education: Theory, research, and practice (pp. 39–62). Jossey‐Bass/Wiley.

[aphw70186-bib-0069] Saberian, S. , Heyes, A. , & Rivers, N. (2017). Alerts work! Air quality warnings and cycling. Resource and Energy Economics, 49, 165–185. 10.1016/j.reseneeco.2017.05.004

[aphw70186-bib-0070] Saelens, B. E. , & Handy, S. L. (2008). Built environment correlates of walking: A review. Medicine and Science in Sports and Exercise, 40, S550–S566. 10.1249/MSS.0b013e31817c67a4.Built Built 18562973 PMC2921187

[aphw70186-bib-0071] Sallis, J. F. , Owen, N. , & Fisher, E. B. (2008). Ecological models of health behavior. In K. Glanz , B. K. Rimer , & K. Viswanath (Eds.), Health behavior and health education: Theory, research, and practice (4th ed.) (pp. 465–485). Jossey‐Bass/Wiley.

[aphw70186-bib-0072] Shehab, M. A. , & Pope, F. D. (2019). Effects of short‐term exposure to particulate matter air pollution on cognitive performance. Scientific Reports, 9 (1), 8237. 10.1038/s41598-019-44561-0 31160655 PMC6546704

[aphw70186-bib-0073] Strusi, C. , Dagliati, A. , Pala, D. , Larizza, C. , Bellazzi, R. , & Quaglini, S. (2022). Taking a walk avoiding polluted routes: an application to a virtual coach for cancer. In MELECON 2022 – IEEE Mediterranean Electrotechnical Conference, Proceedings (pp. 1107–1111). 10.1109/MELECON53508.2022.9843091

[aphw70186-bib-0074] Sun, D. , Liu, C. , Ding, Y. , Yu, C. , Guo, Y. , Sun, D. , Pang, Y. , Pei, P. , Du, H. , Yang, L. , Chen, Y. , Meng, X. , Liu, Y. , Liu, J. , Sohoni, R. , Sansome, G. , Chen, J. , Chen, Z. , Lv, J. , … China Kadoorie Biobank Collaborative Group . (2023). Long‐term exposure to ambient PM2·5, active commuting, and farming activity and cardiovascular disease risk in adults in China: A prospective cohort study. The Lancet. Planetary health, 7 (4), e304–e305. 10.1016/S2542-5196(23)00047-5 37019571 PMC10104773

[aphw70186-bib-0075] Tainio, M. , Jovanovic Andersen, Z. , Nieuwenhuijsen, M. J. , Hu, L. , de Nazelle, A. , An, R. , Garcia, L. M. T. , Goenka, S. , Zapata‐Diomedi, B. , Bull, F. , & Sá, T. H. (2021). Air pollution, physical activity and health: A mapping review of the evidence. Environment International, 147, 105954. 10.1016/j.envint.2020.105954 33352412 PMC7816214

[aphw70186-bib-0076] Timm, I. , Reichert, M. , Ebner‐Priemer, U. W. , & Giurgiu, M. (2023). Momentary within‐subject associations of affective states and physical behavior are moderated by weather conditions in real life: An ambulatory assessment study. International Journal of Behavioral Nutrition and Physical Activity, 20 (1), 117. 10.1186/s12966-023-01507-0 37777773 PMC10541720

[aphw70186-bib-0077] Troiano, R. P. , Berrigan, D. , Dodd, K. W. , Mâsse, L. C. , Tilert, T. , & Mcdowell, M. (2008). Physical activity in the United States measured by accelerometer. Medicine and Science in Sports and Exercise, 40 (1), 181–188. 10.1249/mss.0b013e31815a51b3 18091006

[aphw70186-bib-0078] Trull, T. J. , & Ebner‐Priemer, U. (2013). Ambulatory assessment. Annual Review of Clinical Psychology, 9, 151–176. 10.1146/annurev-clinpsy-050212-185510 PMC4249763 23157450

[aphw70186-bib-0079] Turrisi, T. B. , Bittel, K. M. , West, A. B. , Hojjatinia, S. , Hojjatinia, S. , Mama, S. K. , Lagoa, C. M. , & Conroy, D. E. (2021). Seasons, weather, and device‐measured movement behaviors: A scoping review from 2006 to 2020. International Journal Od Behavioral Nutrition and Physical Activity, 18 (24), 1–26. 10.1186/s12966-021-01091-1 PMC7863471 33541375

[aphw70186-bib-0080] U.S. Environmental Protection Agency . (2025). Progress cleaning the air and improving people's health. U.S. EPA. https://www.epa.gov/clean‐air‐act‐overview/progress‐cleaning‐air‐and‐improving‐peoples‐health.

[aphw70186-bib-0081] Wangsan, K. , Panumasvivat, J. , Usanakul, T. , Sirivoravith, V. , Rojanachai, S. , Zheng, N. , Boontan, C. , & Sapbamrer, R. (2024). Impact of ambient air pollution on physical activity engagement among university students. Frontiers in Public Health, 12, 1488115. 10.3389/fpubh.2024.1488115 39635204 PMC11614755

[aphw70186-bib-0082] Wen, X. J. , Balluz, L. , & Mokdad, A. (2009). Association between media alerts of air quality index and change of outdoor activity among adult asthma in six states, BRFSS, 2005. Journal of Community Health, 34 (1), 40–46. 10.1007/s10900-008-9126-4 18821001

[aphw70186-bib-0083] World Health Organization . (2020). WHO guidelines on physical activity and sedentary behaviour. World Health Organization.

[aphw70186-bib-0084] World Health Organization . (2021). WHO global air quality guidelines: Particulate matter (PM2.5 and PM10), ozone, nitrogen dioxide, sulfur dioxide and carbon monoxide. World Health Organization. 34662007

[aphw70186-bib-0085] World Health Organization . (2022). Global status report on physical activity 2022. World Health Organization.

[aphw70186-bib-0086] World Health Organization . (2023). Ambient (outdoor) air quality and health. https://www.who.int/news‐room/fact‐sheets/detail/ambient‐(outdoor)‐air‐quality‐and‐health.

[aphw70186-bib-0087] Wunsch, K. , Eckert, T. , Fiedler, J. , & Woll, A. (2022). Just‐in‐time adaptive interventions in mobile physical activity interventions—A synthesis of frameworks and future directions. The European Health Psychologist, 22 (4), 834–842.

[aphw70186-bib-0088] Yang, Y. , Goh, K. Y. , Teo, H. H. , & Tan, S. S. L. (2024). The impact of air pollution information on individuals' exercise behavior: Empirical study using wearable and mobile devices data. JMIR mHealth and uHealth, 12, e55207. 10.2196/55207 39255029 PMC11422738

[aphw70186-bib-0089] Yao, J. , Tan, C. S. , Lim, N. , Tan, J. , Chen, C. , & Müller‐Riemenschneider, F. (2021). Number of daily measurements needed to estimate habitual step count levels using wrist‐worn trackers and smartphones in 212,048 adults. Scientific Reports, 11 (1), 1–3. 10.1038/s41598-021-89141-3 33953288 PMC8100112

[aphw70186-bib-0090] Yates, T. , Haffner, S. M. , Schulte, P. J. , Thomas, L. , Huffman, K. M. , Bales, C. W. , Califf, R. M. , Holman, R. R. , McMurray, J. J. , Bethel, M. A. , Tuomilehto, J. , Davies, M. J. , & Kraus, W. E. (2014). Association between change in daily ambulatory activity and cardiovascular events in people with impaired glucose tolerance (NAVIGATOR trial): A cohort analysis. Lancet (London, England), 383 (9922), 1059–1066. 10.1016/S0140-6736(13)62061-9 24361242

[aphw70186-bib-0091] Yoo, G. (2021). Real‐time information on air pollution and avoidance behavior: Evidence from South Korea. Population and Environment, 42, 406–424. 10.1007/s11111-020-00368-0 33191965 PMC7653214

[aphw70186-bib-0092] You, Y. , Wang, D. , Liu, J. , Chen, Y. , Ma, X. , & Li, W. (2022). Physical exercise in the context of air pollution: An emerging research topic. Frontiers in Physiology, 13 (February), 1–21. 10.3389/fphys.2022.784705 PMC8918627 35295574

[aphw70186-bib-0093] Yu, M. , Wu, Y. , Gordon, S. P. , Cheng, J. , Chen, P. , Wang, Y. , & Yu, H. (2021). Objectively measured association between air pollution and physical activity, sedentary behavior in college students in Beijing. Environmental Research, 194 June 2020, 110492. 10.1016/j.envres.2020.110492 33217438

[aphw70186-bib-0094] Zhang, Y. , Ke, L. , Fu, Y. , Di, Q. , & Ma, X. (2022). Physical activity attenuates negative effects of short‐term exposure to ambient air pollution on cognitive function. Environment International, 160, 107070. 10.1016/j.envint.2021.107070 34973588

