# Supplementary material for: Daily relationship between air pollution and physical activity in an industrial region
Source: Appl Psychol Health Well Being. 2026 Jul 7;18(4):e70186. doi: 10.1111/aphw.70186 (PMC13342454; doi:10.1111/aphw.70186)

# Supplementary Materials

## S1. Model equations

Subscripts d and i denote day and individual, respectively.

**Level 1 (Day-level / Within-person model):**

**Daily Step_di_ =** β_0i_ + β_1i_(PM₁₀)_di_ + β_2i_(Temperature)_di_ + β_3i_(Precipitation)_di_ + β_4i_(Sunshine)_di_ + β_5i_(Weartime)_di_ + β_6i_(lockdown)_di_ + e_di_

**Level 2 (Person-level / Between-person model):**

**Random effects:**

- **Random Intercept (with between-person level covariates)**

**β_0i_ =** γ_00_ + γ_01_(PA Status)_i_ + γ_02_(Gender)_i_ + γ_03_(Education)_i_ + γ_04_(Age)_i_ + γ_05_(AP Monitoring)_i_ +

γ_06_(Avg PM₁₀)_i_ + γ_07_(Avg Precipitation)_i_ + γ_08_(Avg Sunshine)_i_ + γ_09_(Avg Weartime)_i_ + γ_010_(Avg PM₁₀ x AP Monitoring)_i_ + u_0i_

- **Random Slope (daily** PM₁₀ **effect on daily steps) with Cross-level Interaction**

**β_1i_ =** γ_10_ + γ_11_(AP Monitoring) _i_ + u_1i_

**Fixed effects:**

- **β_2i_ =** γ_20_
- **β_3i_ =** γ_30_
- **β_4i_ =** γ_40_
- **β_5i_ =** γ_50_
- **β_6i_ =** γ_60_

## S2. Mplus code (simplified)

DATA: file is data.csv;

Variable: NAMES ARE id day steps wear PM10 temp prec sun run male univ age foll PM10_follow lock;

USEVARIABLES ARE id

steps ! Steps: daily step counts
 wear ! Daily Fitbit weartime
 PM10 !daily air pollution
 temp !daily temperature
 prec ! Daily precipitation
 sun ! Daily sunshining hours
 run !run: runner vs. Non-runner

male ! sex

univ ! education

age ! age

foll ! AP monitoring
 PM10_follow ! Interaction term between average PM10 and AP monitoring

lock; ! A binary indicator of whether during the COVID lockdown.

CLUSTER = id;
BETWEEN = run male univ age foll PM10_follow;
WITHIN = lock;
! If a variable is not declared in both Between and Within, latent mean centering is applied.

MISSING = ALL(-999);

Define: CENTER wear PM10 temp prec sun run male age univ foll (GrandMEAN);

ANALYSIS: TYPE IS TWOLEVEL RANDOM;
 ESTIMATOR IS BAYES;
 BITER=(10000); !Minimum number of iterations

MODEL:
**%WITHIN%**
pm10;
RS1| steps on pm10; ! Random slope
steps on temp prec sun wear lock;

pm10 temp prec sun WITH temp prec sun; ! Covariances

**%BETWEEN%**
steps ON run male univ age foll;

steps ON PM10 prec sun wear;
steps ON PM10_follow; ! Is the relationship moderated by checking the AP sta
RS1 ON foll; ! Cross-level moderation

steps WITH RS1;
run WITH wear;

OUTPUT: std tech8;

## S3. Bayesian estimation details

We used Bayesian estimation in Mplus 8 to fit a two-level multilevel model examining the association between daily air pollution (PM₁₀) and physical activity (step counts). The Bayesian estimator used the Markov Chain Monte Carlo (MCMC) algorithm based on the Gibbs sampler (Gelman et al., 1995), with the following specifications and the default settings of Mplus:

- Number of Chains: 2
- Iterations per Chain: 10,000
- Burn-in: 5,000 iterations (per chain)

The default settings include conjugate priors: diffuse normal priors for regression coefficient (mean = 0, variance = 10^10), inverse-gamma priors for residual variances, and Wishart priors for covariance matrices. Mplus uses the potential scale reduction (PSR) criterion for convergence assessment, and a burn-in period consisting of the first half of the iterations for each chain. Point estimates were derived from the median posterior distribution. For more details about the priors, see Asparouhov and Muthén (2010).

**References**

Gelman, A., Carlin, J. B., Stern, H. S., & Rubin, D. B. (1995). *Bayesian Data Analysis.* Chapman & Hall. <https://doi.org/10.1201/9780429258411>

Asparouhov, T., & Muthén, B. (2010). *Bayesian analysis using Mplus: Technical implementation*. <https://www.statmodel.com/download/Bayes2.pdf>

## S4. Simulation-Based Power Analysis

library(lme4)

library(MASS) # for mvrnorm

**# 1. data ----**

dat <- read.csv("AP_data_cleaned_mplus.csv") dat$id <- factor(dat$id)

**# 2. parameters (estimated) ----**

**## Residual variances**

sigma2_within <- 36.839 # Within residual variance

tau2_between <- 15.838 # Between residual variance

sigma_within <- sqrt(sigma2_within)

tau_between <- sqrt(tau2_between)

**## Fixed effects**

beta0 <- 13.747 # intercept

beta_PM10_within <- -0.03 # within-person PM10_CMC

beta_PM10_between <- -0.013 # between-person PM10_CM_GMC

beta_follow <- -0.736 # foll main effect

beta_interaction <- -0.034 # PM10_CMC × follow_GMC

**# 3. Monte Carlo Simulation ----**

set.seed(123)

nsim <- 1000

N <- nrow(dat)

ids <- unique(dat$id)

J <- length(ids)

sig_within <- logical(nsim)

sig_between <- logical(nsim)

sig_xlevel <- logical(nsim)

for (s in 1:nsim) {

**## Random effect variance & covariance matrix**

Sigma_u <- matrix(c(15.838, -0.198, -0.198, 0.024), nrow = 2, byrow = TRUE)

**## for each person (u0, u1) ~ MVN(0, Sigma_u)**

u_mat <- mvrnorm(J, mu = c(0, 0), Sigma = Sigma_u)

u0_j <- u_mat[, 1]

u1_j <- u_mat[, 2]

u0_i <- u0_j[match(dat$id, ids)]

u1_i <- u1_j[match(dat$id, ids)]

**## estimates**

eta <- beta0 + (beta_PM10_within + u1_i) * dat$PM10_CMC + beta_PM10_between * dat$PM10_CM_GMC + beta_follow * dat$follow_GMC + beta_interaction * dat$PM10_CMC * dat$follow_GMC + u0_i

**## add residual noise**

y_sim <- eta + rnorm(N, mean = 0, sd = sigma_within)

**## fit models**

fit_sim <- lmer( y_sim ~ PM10_CMC + PM10_CM_GMC + follow_GMC + PM10_CMC:follow_GMC + (1 + PM10_CMC | id), data = dat, REML = TRUE )

coefs <- summary(fit_sim)$coef

**## test each effect**

z_within <- coefs["PM10_CMC", "Estimate"] / coefs["PM10_CMC", "Std. Error"]

z_between <- coefs["PM10_CM_GMC", "Estimate"] / coefs["PM10_CM_GMC", "Std. Error"] z_xlevel <- coefs["PM10_CMC:follow_GMC", "Estimate"] / coefs["PM10_CMC:follow_GMC", "Std. Error"]

sig_within[s] <- abs(z_within) > 1.96

sig_between[s] <- abs(z_between) > 1.96

sig_xlevel[s] <- abs(z_xlevel) > 1.96

}

**# power ----**

power_within <- mean(sig_within)

power_between <- mean(sig_between)

power_xlevel <- mean(sig_xlevel)

power_within

power_between

power_xlevel

## S5. Examination of nonlinear effects of PM₁₀

To evaluate whether the association between daily PM₁₀ concentrations and daily step counts exhibited a nonlinear form, we conducted generalized additive models (GAMs) using the *mgcv* package in R. These models estimate the effect of PM₁₀ using penalized spline smoothing, allowing the shape of the association to be data-driven rather than restricted to a linear form. The models included the same daily covariates as in the primary analysis (temperature, precipitation, sunshine, weekday, and wear time), ensuring that any remaining curvature in the PM₁₀–steps relationship was not attributable to confounding by other weather-related factors.

Across all specifications, the estimated smooth term for PM₁₀ had effective degrees of freedom near 1.0, indicating that the spline reduced to a linear function. The nonlinear component was not statistically significant (e.g., F = 1.53, p = .217), and visual inspection of the smooth term confirmed the absence of meaningful curvature across the observed PM₁₀ range. The full GAM results and a plot of the smooth function are provided in Figure below.


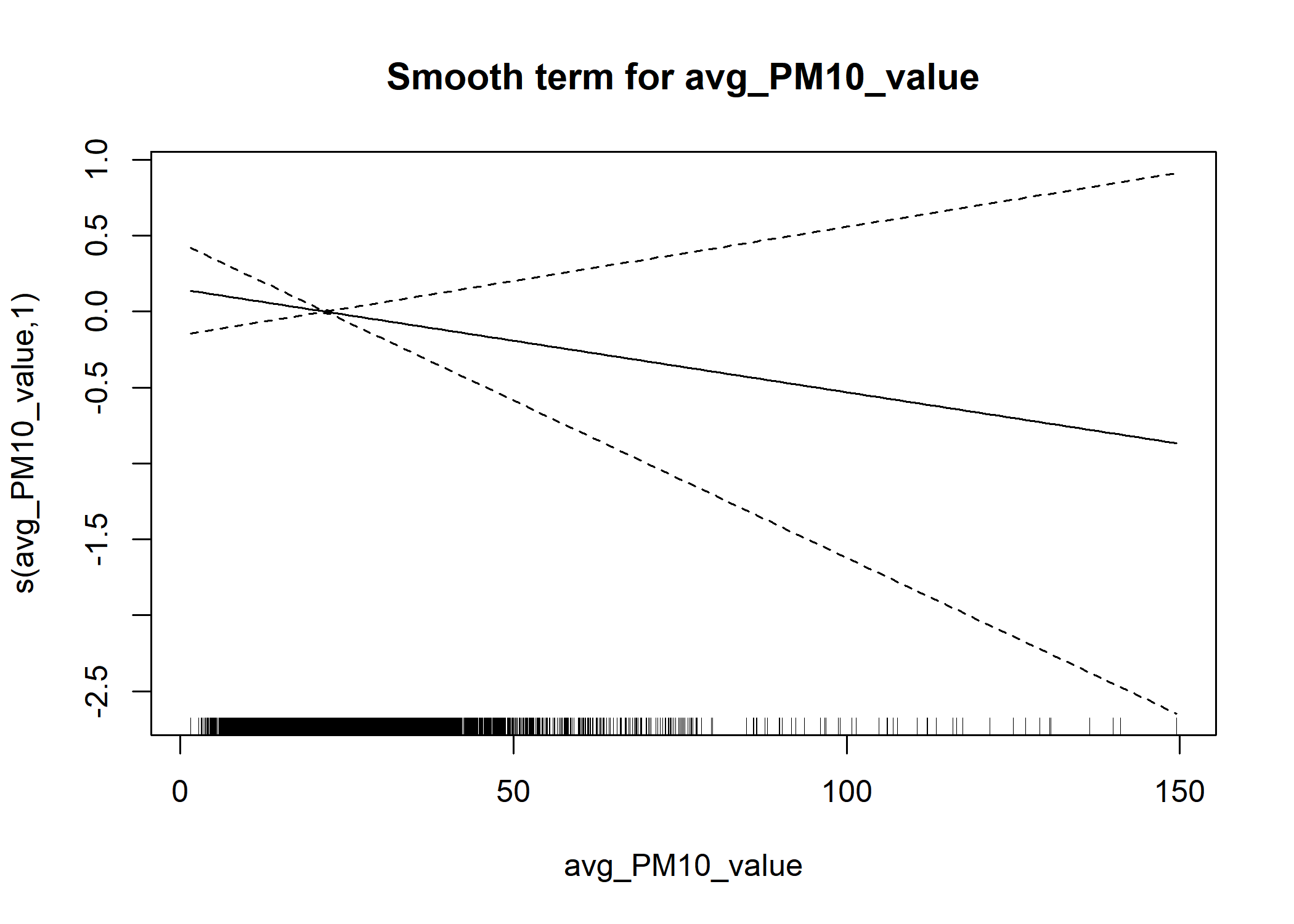


These supplementary analyses support the conclusion that the PM₁₀–steps relationship is well approximated by a linear form, consistent with the linear specification used in the multilevel Bayesian model.

## S6. Model assumptions check


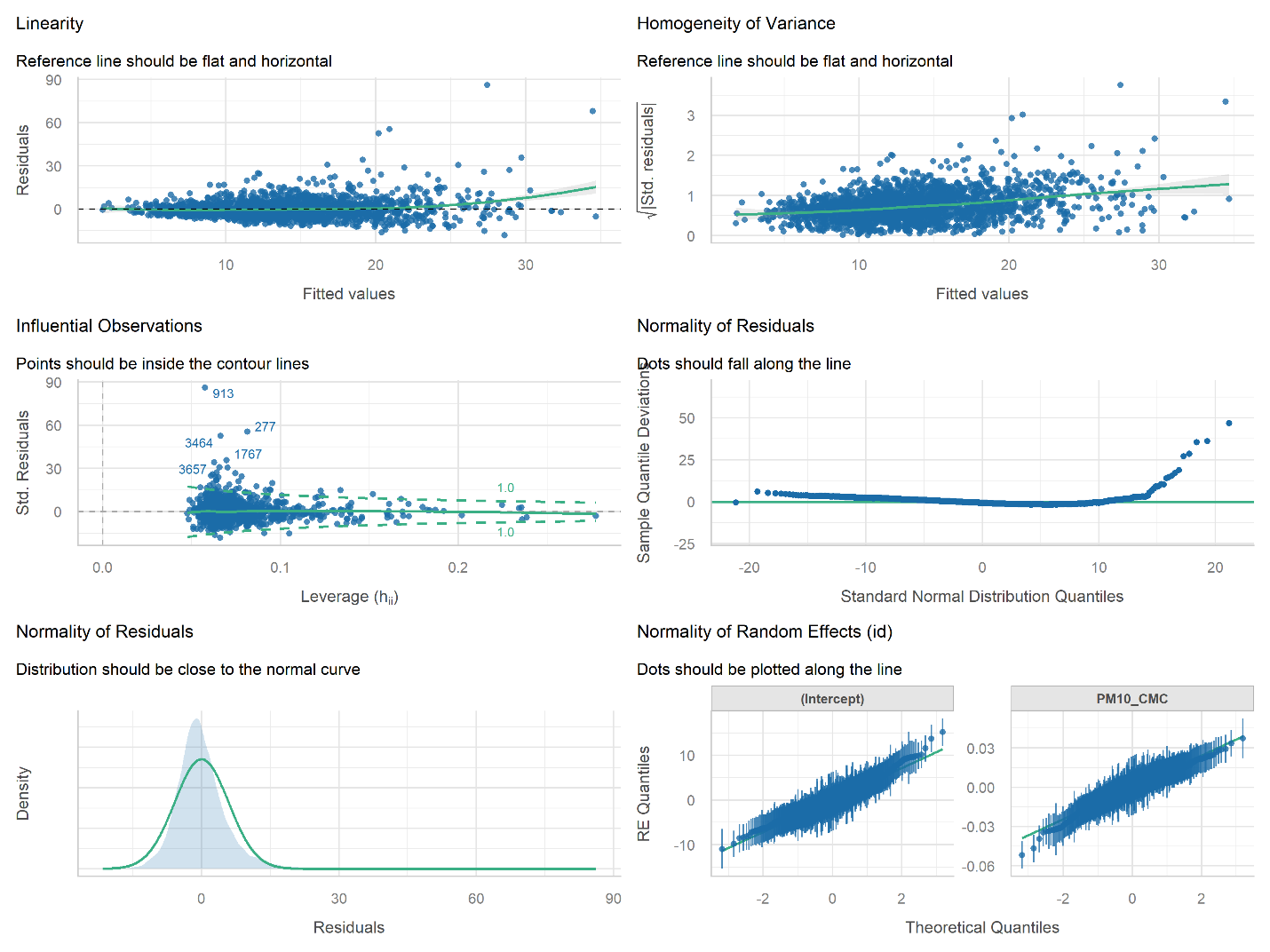


We have conducted and reported comprehensive model assumption checks using check_model() function of R package performance (Lüdecke et al., 2021). Specifically, we evaluated linearity, homoscedasticity, normality of residuals, and the distribution of random effects. Diagnostic plots (residuals vs. fitted values, QQ plots, leverage and influence plots, residual density plots, and random effect QQ plots) indicated that the model assumptions were adequately met. Residuals showed no systematic departures from linearity, only mild heteroscedasticity without a funnel-shaped pattern, and an acceptable degree of normality. Random intercepts and slopes also followed approximately normal distributions.

**References**

Lüdecke, D., Ben-Shachar, M., Patil, I., Waggoner, P., & Makowski, D. (2021). Performance: An R package for assessment, comparison and testing of statistical models. *Journal of Open Source Software, 6*(60), 3139. <https://doi.org/10.21105/joss.03139>

## S7. Location-based data – technical appendix

To reliably approximate participants’ environmental exposure while preserving their privacy, we constructed a location-proxy system that linked smartphone-derived positional information to the nearest air-quality monitoring stations. Smartphone location was obtained by the smartphone study survey app, when the participant interacted with the app (e.g., completed an EMA survey), using the device’s native OS location services (Core Location for iOS and Location API for Android). The app assigned the event to the geographically closest station within the National Air Quality Monitoring Network (NAQMN), operated Czech Hydrometeorological Institute (CHMI), and recorded the timestamp, the longitude and latitude of the nearest station and distance to it from the actual location of the participant. This process did not involve storing participants’ precise location (adhering to the study procedures approved by the ethics committee), thereby protecting privacy while still capturing approximate location information.

Although operational data from all NAQMN stations were available in near-real time and periodically stored by the researchers, the nearest monitoring station locations and their operational data consisted of point-in-time observations rather than continuous records. These data correspond only to the specific, non-periodic timestamps (at an hourly resolution) recorded when the survey app obtained the smartphone location, which varied based on participant activity and device settings. Because these point-in-time data lack a consistent 24-hour profile, they cannot be aggregated into daily averages. Furthermore, a detailed inspection revealed frequent missing data at specific stations due to routine instrument calibration, temporary outages, scheduled maintenance, or a focus on a subgroup of air pollution parameters. Moreover, the stored data had not yet undergone the annual validation process, typically completed by the CHMI by July 1 of the following year. The nearest monitoring stations also included traffic and industrial sites, which typically exhibit very limited spatial representativeness (up to 10–100 m) because their measurements are heavily influenced by proximate emission sources (EEA, 1999). Since location sampling was neither periodic nor continuous by study design, these nearest monitoring station locations provide valuable information on the approximate locations of participants on a given day, but they do not provide full or exact spatiotemporal coverage of their whereabouts.

To address these issues, we used the nearest-station locations solely as a proxy for participant locations and linked them with data from the nearest background air pollution monitoring station and weather station. This approach enabled us to connect each location proxy record with validated daily air-quality and weather data from Open Data CHMI (CHMI, 2025; described in Section 2.3.3 Location-based data). To ensure data quality and maintain spatial consistency, all records were subjected to filtering based on location information accuracy and distance thresholds. Specifically, we required:

1. a positional accuracy of ≤10 km, which reflects the approximate city-level location accuracy achievable in in low-power mode of the native location services (Significant-Change Location Service within Core Location or PRIORITY_LOW_POWER priority constant for Location API; Apple, 2026; Google, 2026);
2. a maximum distance of ≤40 km from the participant to the nearest station and subsequently to the nearest representative background station, a value determined from spatial analyses of the NAQMN network layout and the approximate coverage area of largest background sites (see nearest-station service areas in Figure 2 of the main article);
3. a maximum daily participant relocation threshold of ≤80 km, reflecting the legislatively defined spatial service area of the NAQMN network (Act No. 201/2012 Coll. Act on Air Protection), where one monitoring location covers roughly 20,000 km² (approximately a 79.79 km radius) in polluted regions of Czech Republic.

These constraints were applied to minimize potential misclassification of participant exposure arising from low-precision location estimates or atypically large daily relocations, which would not allow for a sufficiently valid assignment of daily air pollution exposure values.

**References**

Apple. (2026). *Core Location*. Apple Developer Documentation. <https://developer.apple.com/documentation/corelocation>

Czech Republic. (2012). *Act No. 201/2012 Coll., on Air Protection*. Collection of Laws of the Czech Republic.

Czech Hydrometeorological Institute. (2025). *Open data on air quality, hydrology and meteorology* [Data set]. <https://opendata.chmi.cz/>

European Environment Agency. (1999, April 14). *Criteria for EUROAIRNET: The EEA air quality monitoring and information network* (Technical report No. 12). <https://www.eea.europa.eu/publications/TEC12/>

Google. (2026, March 30). *Change location settings — Sensors and location*. Android Developers. <https://developer.android.com/develop/sensors-and-location/location/change-location-settings>

S8. Monthly distributions of daily PM₁₀ concentrations across all study background stations


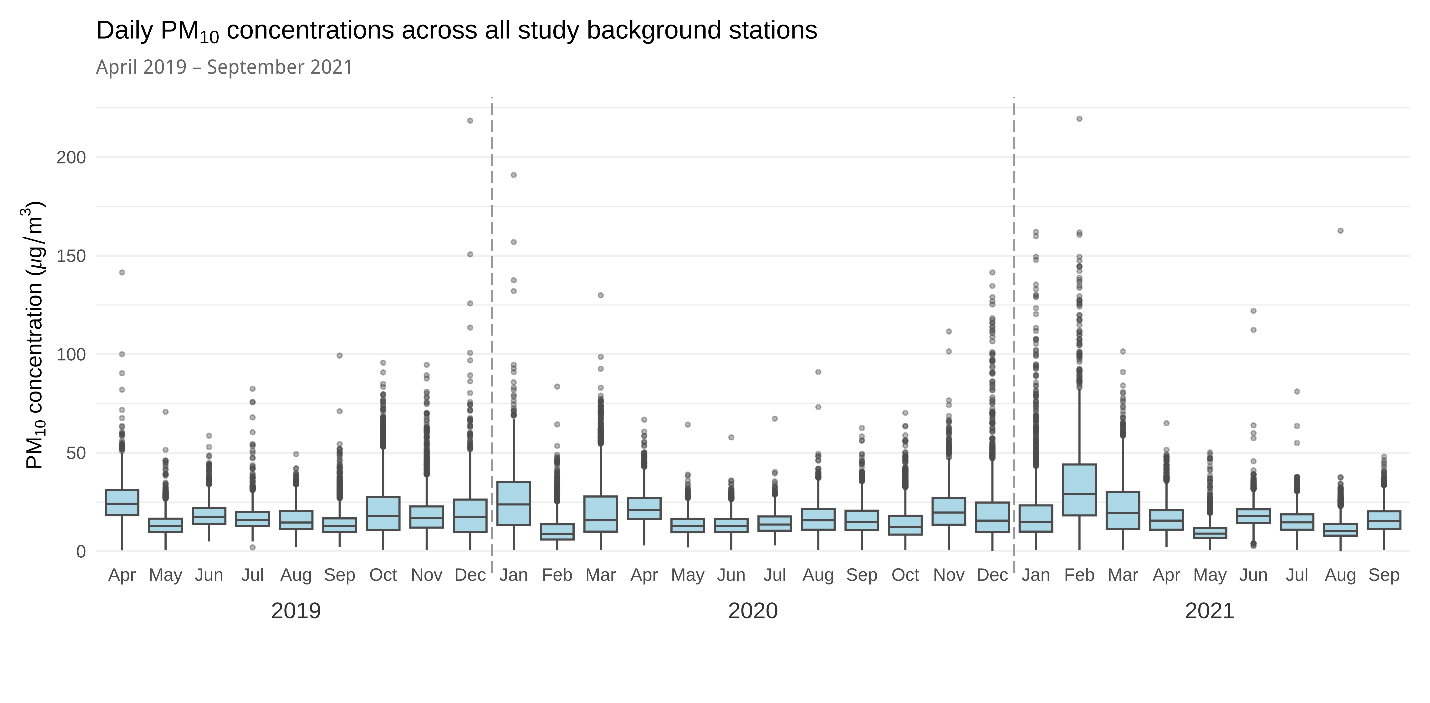

Supplement: Supplementary file 1 — Data S1. Model equations. Full specification of the two‐level multilevel model, including within‐person and between‐person equations, random effects, and cross‐level interactions. Data S2. Mplus code. Simplified Mplus syntax used for estimating the multilevel Bayesian model. Data S3. Bayesian estimation details. Description of the Bayesian estimation procedure, prior distributions, Markov Chain Monte Carlo settings, convergence assessment, and estimation criteria. Data S4. Simulation‐based power analysis. Monte Carlo simulation code and procedures used to evaluate statistical power. Data S5. Examination of nonlinear effects of PM₁₀. Generalized additive model analyses assessing potential nonlinear associations between daily PM₁₀ concentrations and daily step counts, including supplementary results and figures. Data S6. Model assumptions check. Diagnostic analyses evaluating linearity, homoscedasticity, normality of residuals, and random‐effects distributions, with accompanying diagnostic plots. Data S7. Location‐based data – technical appendix. Detailed description of the location‐proxy methodology, linkage of participant locations to air‐quality monitoring stations, exposure assignment procedures, privacy safeguards, and data‐quality filtering criteria. Data S8. Monthly distributions of daily PM₁₀ concentrations across all study background stations. Supplementary figure presenting monthly distributions of PM₁₀ concentrations during the study period. [file APHW-18-0-s001.docx]
